# Spatio-temporal trends of artemisinin-based combination therapy efficacy from 2010 to 2024 in sub-Saharan Africa: a systematic review and meta-analysis

**DOI:** 10.1186/s12879-025-12130-8

**Published:** 2025-11-25

**Authors:** Francis Emmanuel Towanou Bohissou, Guétawendé Job Wilfried Nassa, Paul Sondo, Toussaint Rouamba, Juliana Inoue, Berenger Kaboré, Victor Asua, Jana Held, Halidou Tinto

**Affiliations:** 1 Institute of Tropical Medicine, University Hospital Tübingen, 72074 Tübingen, Germany; 2 Institut de Recherche en sciences de la Santé (IRSS)/Clinical Research Unit of Nanoro (CRUN), Nanoro, Burkina Faso; 3 Centre de Recherche Entomologique de Cotonou (CREC), Cotonou, Benin; 4 Infectious Diseases Research Collaboration (IDRC), Kampala, Uganda; 5 German Center for Infection Research (DZIF), Partner Site Tübingen, Tübingen, Germany; 6 Centre de Recherches Médicales de Lambaréné, (CERMEL), Lambaréné, Gabon

**Keywords:** ACT, Efficacy, Sub-Saharan Africa, Systematic review, Meta-analysis

## Abstract

**Background:**

Artemisinin-based Combination Therapy (ACT) has contributed to the reduction of malaria burden in sub-Saharan Africa. However, the number of global cases has risen since 2015. Resistances to artemisinin reported from Southeast Asia and recently emerged in sub-Saharan Africa might threaten ACT efficacy. We conducted a systematic review and meta-analysis on ACT efficacy trends in sub-Saharan Africa from January 2010 to December 2024.

**Methods:**

We systematically searched PubMed/Medline and Scopus for studies published between 2010 and 2024 that met the World Health Organisation (WHO) criteria for therapeutic efficacy studies. Two reviewers have independently assessed the eligibility criteria and extracted data. ACT efficacy was measured using the PCR-corrected Adequate Clinical and Parasitological Response (ACPR) at day 28 or 42. Meta-analysis was conducted using R.

**Results:**

The meta-analysis included 116 studies with a total of 17,341 participants for artemether-lumefantrine (AL), 8,855 for artesunate-amodiaquine (AS-AQ), 5,544 for dihydroartemisinin-piperaquine (DHA-PPQ), and 346 for artesunate-pyronaridine (AS-PY). Over the period under review, from 2010 to 2024, the PCR-corrected ACPR for AS-AQ, DHA-PPQ, and AS-PY remained above 90% across sub-Saharan Africa. For AL, the PCR-corrected ACPR remained high between 2010 and 2014, consistently exceeding 90% (range: 91–100%). However, from 2015 to 2024, the efficacy showed greater variability, with PCR-corrected ACPR values ranging from 74% to 100%. Notably, this efficacy dropped below the 90% threshold in several countries, including Kenya (2017), Burkina Faso (2018), Uganda (2019), and Nigeria (2020).

**Conclusions:**

While AS-AQ, DHA-PPQ, and AS-PY have maintained high efficacy over time in sub-Saharan Africa, there is a concern about the declining efficacy of AL in some West and East African countries. Our findings suggest that AS-PY could be a promising candidate for inclusion in first-line malaria treatments to address the declining efficacy of AL. Continuous monitoring of ACT efficacy, innovative and efficient control strategies are crucial to prevent the spread of antimalarial drug resistance.

**Registration:**

PROSPERO number CRD42023432718

**Supplementary information:**

The online version contains supplementary material available at 10.1186/s12879-025-12130-8.

## Background

Malaria is a leading parasitic disease that poses a significant public health challenge due to its high mortality rate, largely associated with *Plasmodium falciparum* infections. According to the World Malaria Report 2024, there were an estimated 263 million malaria cases worldwide in 2023, 94% of them occurring in the World Health Organization (WHO) African Region [1]. Moreover, malaria-related deaths were estimated at 597,000, with 95% of them occurring in sub-Saharan Africa. However, a gradual reduction in malaria mortality has been observed, with a decrease of the number of deaths from 622,000 in 2020 to 597,000 in 2023 [1]. This decline is likely attributed to the implementation of various strategic control interventions in endemic countries.

Strategies to control malaria include diagnosing and promptly, effectively treating confirmed cases. Since 2005, the widespread use of ACTs has played an important role in reducing malaria cases and preventing progression to severe malaria, while intravenous artesunate, an artemisinin derivate has been crucial in decreasing malaria-related mortality. Currently, the WHO recommends six ACTs for the treatment of uncomplicated malaria: artemether-lumefantrine (AL), artesunate-amodiaquine (AS-AQ), dihydroartemisinin-piperaquine (DHA-PPQ), artesunate+sulfadoxine-pyrimethamine (AS+SP), artesunate-mefloquine (AS-MQ), and artesunate-pyronaridine (AS-PY) [1, 2]. In 2023, national malaria control programmes distributed 235 million doses of ACTs, predominantly in sub-Saharan Africa [1]. AL remains the most widely used ACT, followed by AS-AQ and DHA-PPQ, which serve as the first-line treatments in many countries. AS-PY recently introduced in 2023, is part of the antimalarial drug policy only in Cameroon, Burkina Faso, the Democratic Republic of the Congo (DRC), and Nigeria, while AS-MQ is included in São Tomé and Príncipe [1].

Partial resistance of *Plasmodium falciparum* to artemisinin was first documented in Southeast Asia in 2008 [3–5]. However recent genomic studies have shown that *PfKelch13* mutations associated with partial artemisinin resistance have independently emerged in several African countries [1, 5–9]. These mutations often coexist with long-established variants conferring resistance to other antimalarials, such as chloroquine (*Pfcrt*) and sulfadoxine-pyrimethamine (*Pfdhfr* and *Pfdhps*), highlighting the complex multidrug-resistant background of parasite populations [10–13]. Surveillance has also reported a rising prevalence of WHO-validated *PfKelch13* candidate mutations—including R561H, A675V, R622I, and C469Y—across countries such as Rwanda, Tanzania, Uganda, Ethiopia, Sudan, and Eritrea [14–19]. Importantly, these genetic changes are accompanied by observed declines in the efficacy of artemether–lumefantrine (AL), with some studies reporting efficacy rates below 90%, e.g. in Angola [20], the Democratic Republic of the Congo (DRC) [21], Burkina Faso [22], and Uganda [23].

To early detect resistance to artemisinin derivatives and their partner drugs, the WHO recommends the monitoring of ACTs’ efficacy in malaria-endemic regions [24]. Studies conducted in sub-Saharan African countries, including Kenya, Uganda, and Angola, reported reduced parasite clearance rates and increased cases of recrudescence a few years after the introduction of AL and DHA-PPQ [25–27]. Delayed parasite clearance time is a key clinical indicator that defines artemisinin partial resistance, highlighting reduced parasite susceptibility and raising significant concerns about the continued efficacy of ACTs in malaria treatment. This resistance contributes to the growing malaria burden and presents a real threat to global malaria control efforts. It highlights the urgent need for continuous monitoring and timely interventions to prevent the further spread of the resistance across the continent.

Systematic reviews on the efficacy of antimalarial drugs have varied in scope, with some studies providing global estimates of drug efficacy [28, 29]. In contrast, others, though they have focused on specific regions, such as sub-Saharan Africa, target either a single country or specific ACTs like AL, DHA-PPQ, and AS-AQ [30–34]. The most recent systematic review and meta-analysis by Marwa et al., published in 2022, examined the therapeutic efficacy of AL, AS-AQ, and DHA-PPQ in sub-Saharan Africa, pooling data from studies conducted between 2010 and 2018 [31]. Recent studies have reported a decline in AL efficacy across sub-Saharan Africa. However, to our knowledge, no study has systematically evaluated temporal trends in ACT efficacy across Africa to monitor changes following the widespread implementation of these therapies. In this systematic review and meta-analysis, we present the spatiotemporal trends in the efficacy of ACTs across sub-Saharan Africa from 2010 to 2024.

## Methods

### Study protocol registration

Studies included in this systematic review and meta-analysis were screened using the Preferred Reporting Items for Systematic Reviews and Meta-Analyses (PRISMA) statement [35]. The study protocol was developed and registered in PROSPERO under the number CRD42023432718. The review protocol was divided into two sections: one focused on the efficacy of ACTs and the second on molecular markers of malaria drug resistance. However, this report will focus on the findings from the efficacy studies.

### Search strategy

The literature search used several databases, including PubMed, EMBASE, African Journals Online, Scopus, and Web of Science. The focus was on studies with sample collections occurring from January 2010 through December 2024. Prior to the final analysis, the searches were updated to identify and retrieve any newly published studies, ensuring that the most recent research was considered for inclusion. The research words used were: Antimalarials – Malaria drug – Artemisinin – Artesunate – Artemether – Lumefantrine – Amodiaquine – Sulfadoxine – Pyrimethamine – Dihydroartemisinin – Piperaquine – Mefloquine – Pyronaridine - *Plasmodium* – *Plasmodium falciparum* – Malaria – Paludisme - sub-Saharan Africa - Efficacy – treatment – treatment failure – effectiveness.

### Inclusion criteria

All studies with samples collected from January 2010 to December 2024 that evaluated the efficacy of at least one recommended ACT for the treatment of uncomplicated *Plasmodium falciparum* malaria in sub-Saharan Africa were reviewed. Therefore, only studies that recruited participants from 2010 onward were included in this analysis. This review assesses the temporal trends in drug efficacy within each country five years after the clinical adoption of these treatments. The decline of the efficacy of antimalarial drugs is associated with the resistance of *P. falciparum*, which is driven by the selection pressure that results from prolonged or suboptimal drug use [34]. The outcome analysed in these studies was the Polymerase Chain Reaction (PCR)-adjusted Adequate Clinical and Parasitological Response (ACPR).

### Exclusion criteria

We excluded the following types of studies: those involving pregnant women or patients with severe malaria, studies conducted before 2010, studies on preventive treatments such as chemoprevention or mass drug administration, research on severe malaria treatment, in vitro or ex vivo studies, studies lacking genotyping results, studies on mixed *Plasmodium* species infections, review papers, volunteer infection studies, non-primary research studies, conference abstracts, studies using artemisinin monotherapy, trials focused solely on safety, trials comparing three-day versus five-day dosing regimens, studies with unsupervised treatment, and research conducted outside sub-Saharan Africa.

### Data extraction

All search results were exported to the Rayyan application (https://rayyan.qcri.org) [36] for review. Two independent reviewers performed the screening process. Following the removal of duplicate records, the reviewers assessed titles and abstracts to identify studies that met the inclusion criteria. Discrepancies were resolved through discussion. Articles deemed eligible during the initial screening underwent a full-text review, with any disagreements addressed before the data extraction. The latter was conducted using Covidence (https://app.covidence.org/reviews) [37], where one reviewer performed the extraction, and the second reviewer verified the data.

### Quality assessment

The methodological quality of the studies was assessed using the National Institute of Health (NIH) study quality assessment tools for controlled intervention studies [38] and was adapted to the WHO TES guidelines [24]. The NIH tool utilized a scoring scale ranging from 0 to 14, with each criterion assigned a value of 1, resulting in a maximum score of 14. These scores were subsequently converted into percentages corresponding to the risk of bias assessment. A score range of 0–60% was classified as low quality, 61–80% as good quality, and 81–100% as excellent quality.

### Data analysis

The efficacy outcomes from the per-protocol analysis of each included study were extracted. In this review, the primary efficacy endpoint following ACT treatment was the ACPR, corrected to exclude new infections using PCR at day 28 or day 42, as recommended by the WHO for the respective ACT. For studies conducted at multiple sites within a country in the same year, the corrected ACPR values were pooled across all sites. Descriptive statistics were used to summarise the synthesised data, including simple counts, ranges, and percentages. Trends in efficacy were assessed by analysing the PCR-corrected ACPR per year and per country using forest plots for each ACT A meta-analysis was conducted using R software to generate the PCR-corrected ACPR with corresponding 95% confidence intervals. Heterogeneity among studies was assessed using Cochran’s Q test and the I^2^ statistic [39]. According to the WHO recommendations, the corrected ACPR ≥90% on day 28 (D28) or 42 (D42) was considered in the analysis, indicating no evidence of resistance to artemisinin or its partner drug [24] that necessitates a policy change.

## Results

### Literature search and study characteristics

**Fig. 1:**
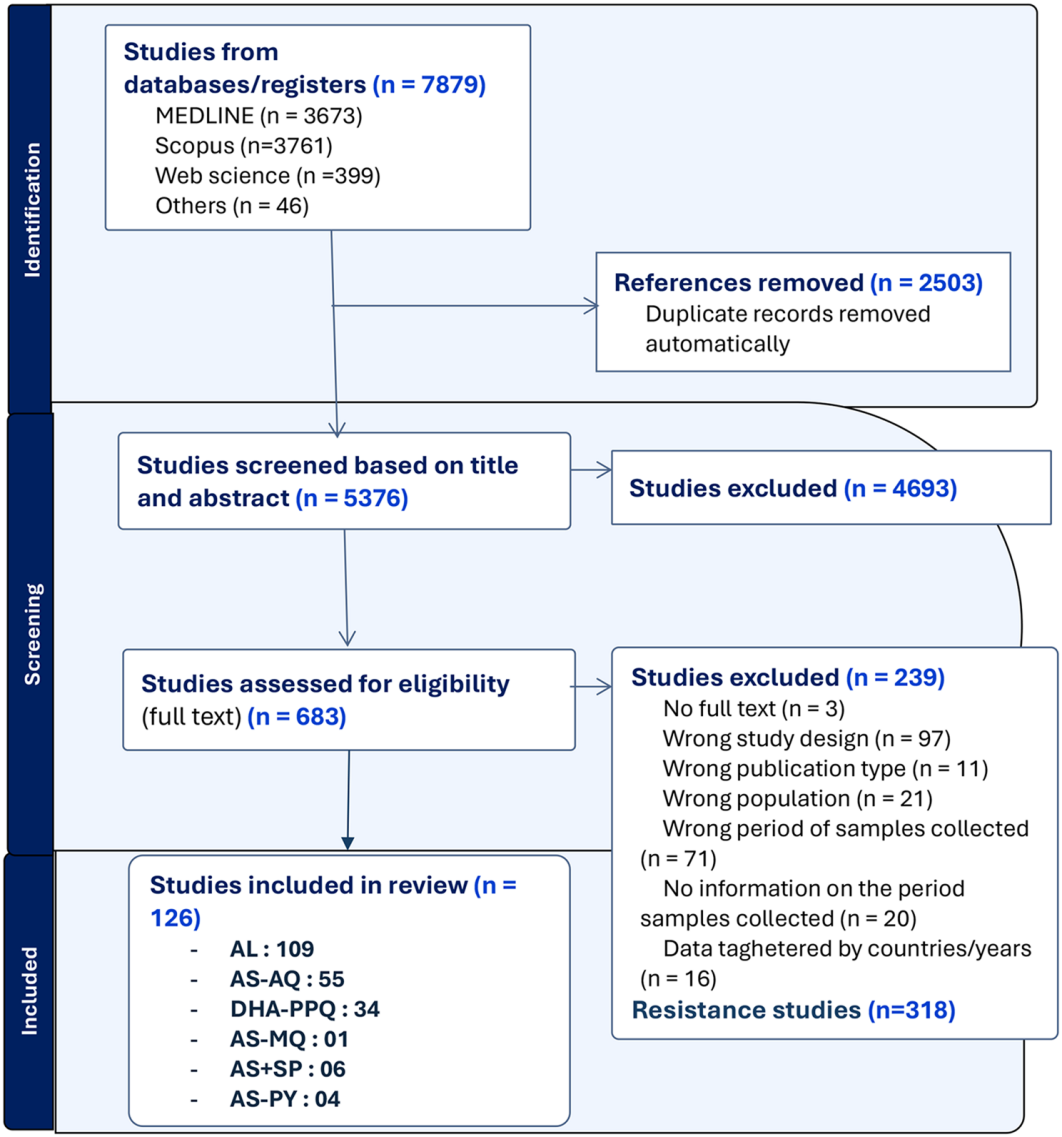
PRISMA flow diagram for article search and screening

A systematic search of electronic databases identified 7,879 studies, from which 683 articles were selected for full-text review. Of these, 444 studies met the criteria for data extraction, and 126 were included in the efficacy analysis (Fig. 1, PRISMA flow diagram). Most of the studies focused solely on AL, accounting for 33.3% (42/126), followed by those involving two-arm AL + AS-AQ (25.4%, 32/126) and AL + DHA-PPQ (12.7%, 16/126). (See Additional File 1, and Supplementary Table 1 of Additional File 2).

**Fig. 2:**
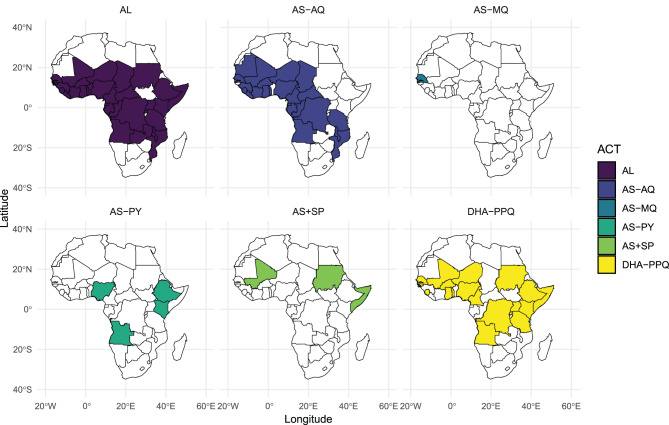
Countries where ACT studies were performed across sub-Saharan Africa from 2010 to 2024 (AL: Artemether Lumefantrine, AS-MQ: Artesunate Mefloquine, AS-PY: Artesunate Pyronaridine, AS-AQ: Artesunate Amodiaquine, AS+SP: Artesunate + Sulfadoxine Pyrimethamine, DHA-PPQ: Dihydroartemisin Piperaquine)

In total, 109 studies on AL were conducted in 32 countries across sub-Saharan Africa, while 55 studies on AS-AQ were conducted in 23 countries, mainly in Central and Western Africa. Regarding DHA-PPQ, 34 studies from 19 countries were included, with the majority conducted in Eastern and Western Africa. A small number of studies (6) investigated AS+SP, mainly in Sudan and Somalia. Four studies on AS-PY were conducted in Nigeria, Angola, Ethiopia, and Kenya (Fig. 2, and See Supplementary Table 2, Additional File 2). The distribution of studies by country revealed that the number of studies from West and Central Africa ranged from 1 to 8 per country, whereas in Eastern Africa, this range was between 7 and 18. Tanzania was represented by 18 studies included in this review (See Supplementary Table 3 and Supplementary Figure 1, Additional File 2).

The risk of bias assessment ranged from 71% to 98%, with no studies classified as low quality. However, 10 studies were excluded from the meta-analysis because they lacked PCR analysis results, or the administration of ACT was unsupervised by investigators. In total, 116 studies were included in the meta-analysis (See Additional File 1).

### PCR-corrected ACPR forest plot analysis per act

**Fig. 3:**
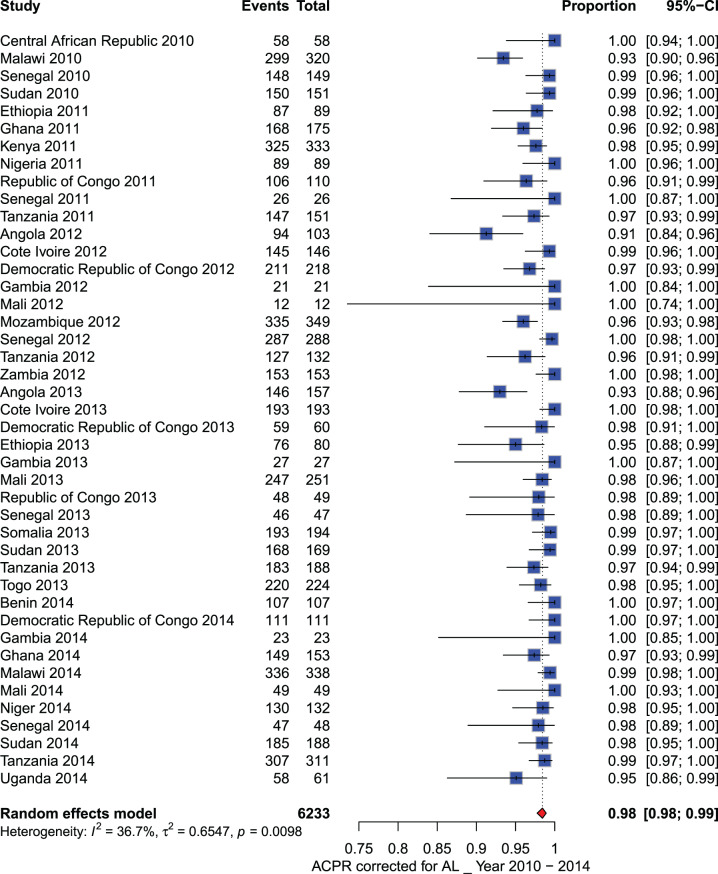
Forest plot for artemether-lumefantrine (AL) ACPR-PCR adjusted based on the per-protocol analysis from 2010 to 2014 in sub-Saharan Africa

**Fig. 4:**
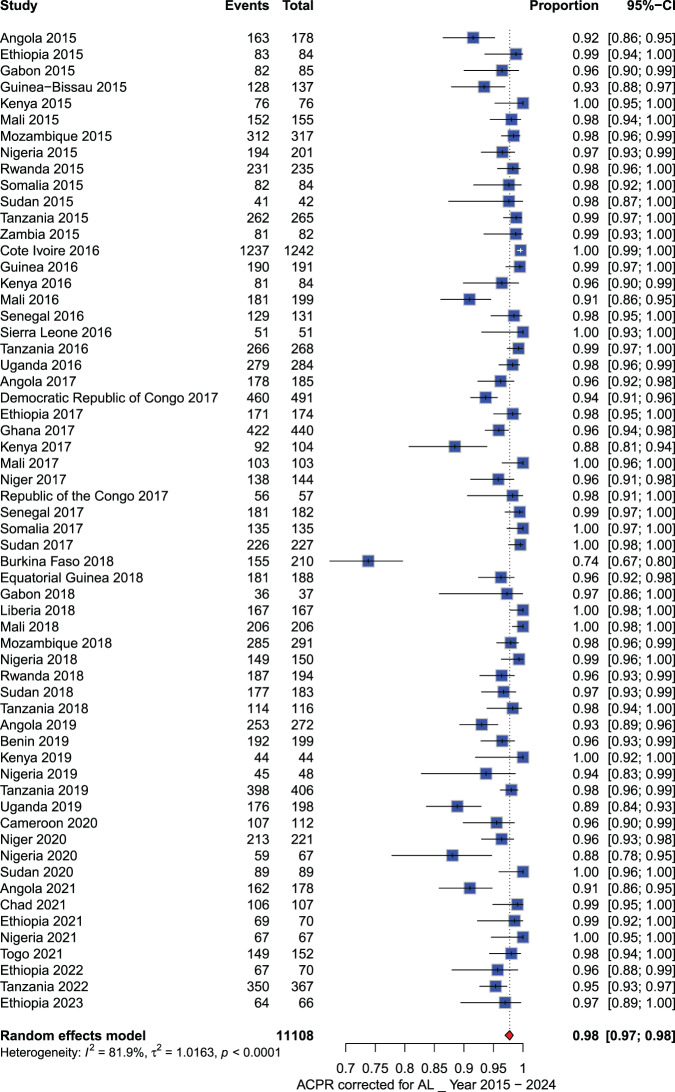
Forest plot for artemether-lumefantrine (AL) ACPR-PCR adjusted based on the per-protocol analysis from 2015 to 2024 in sub-Saharan Africa

**Artemether - lumefantrine (AL):** A total of 17,341 participants were included in the meta-analysis conducted across Africa between 2010 and 2023. From 2010 to 2014, all PCR-corrected ACPR rates at D28 reported in sub-Saharan African countries were above 90%, ranging from 91% to 100%. However, from 2015 to 2023, PCR-corrected ACPR rates ranged from 74% to 100%. Specifically, PCR-corrected ACPR rates below 90% were reported in Kenya (2017), Burkina Faso (2018), Uganda (2019), and Nigeria (2020), with estimates of 88% (92/104), 74% (155/210), 89% (176/198), and 88% (59/67), respectively (See Figs. 3 and 4). Additionally, a study performed in three sites (Benguela, Lunda Sul, and Zaire) in Angola in 2021 showed a decline in AL efficacy observed only in one site (Zaire) (PCR-corrected ACPR at day 28: 88.0%, 95% CI: 82–95%).

**Fig. 5:**
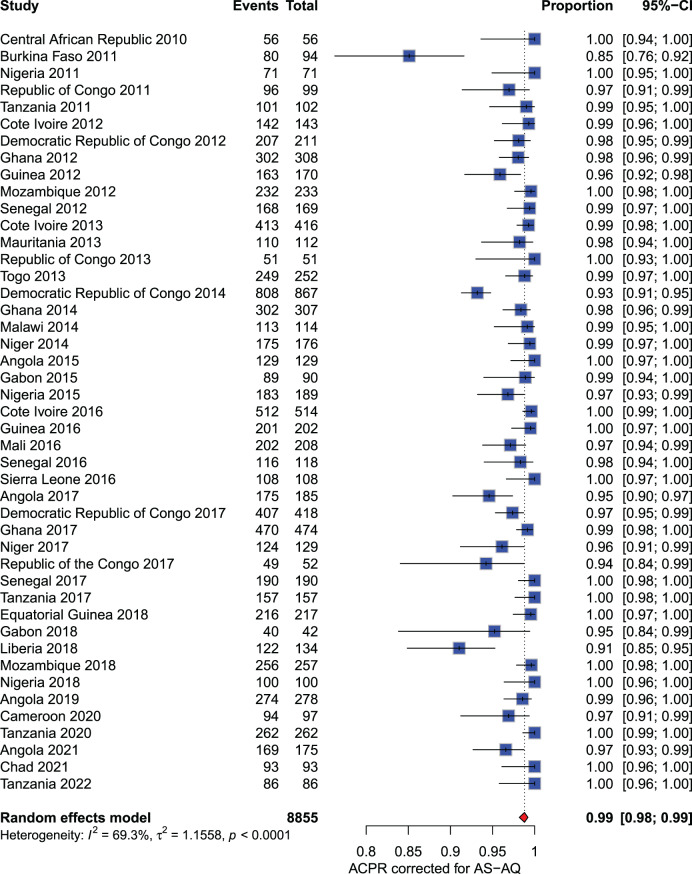
Forest plot for artesunate-amodiaquine (AS-AQ) ACPR-PCR adjusted based on the per-protocol analysis in sub-Saharan Africa from 2010 to 2024

**Artesunate - amodiaquine (AS-AQ):** The meta-analysis included 8,855 participants from studies conducted between 2010 and 2022. Except for Burkina Faso in 2011, where the PCR-corrected ACPR rate at D28 was 85% (80/94), all other PCR-corrected ACPR rates exceeded 90%, ranging from 91% to 100% (Fig. 5).

**Fig. 6:**
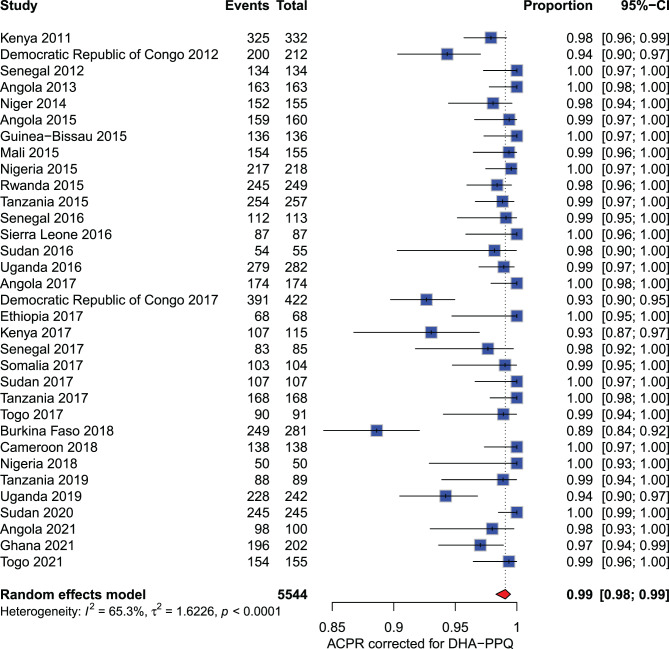
Forest plot for dihydroartemisinin-piperaquine (DHA-PPQ) ACPR-PCR adjusted based on the per-protocol analysis in sub-Saharan Africa from 2010 to 2024

**Dihydroartemisinin - piperaquine (DHA-PPQ):** The meta-analysis included 5,544 participants from 2011 to 2021. Overall, the PCR-corrected ACPR rates at D42 across various countries during this period were consistently above 90%, ranging from 93% to 100%. The only exception was Burkina Faso in 2018, where a PCR-corrected ACPR rate of 89% (249/281) was reported (Fig. 6).

**Fig. 7:**
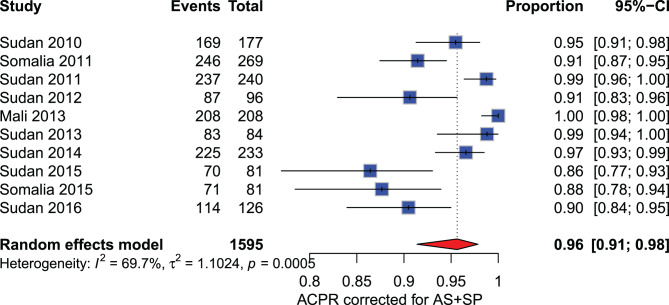
Forest plot for artesunate+sulfadoxine-pyrimethamine (AS+SP) ACPR-PCR adjusted based on the per-protocol analysis in sub-Saharan Africa from 2010 to 2024

**Artesunate - pyronaridine (AS-PY):** The PCR-corrected ACPR rates at D28 among the 346 participants from Kenya (2016), Nigeria (2020), Angola (2021) and Ethiopia (2021) included in the meta-analysis from 2016 to 2021 were consistently high, ranging from 97% to 99% (Fig. 7).

**Fig. 8:**
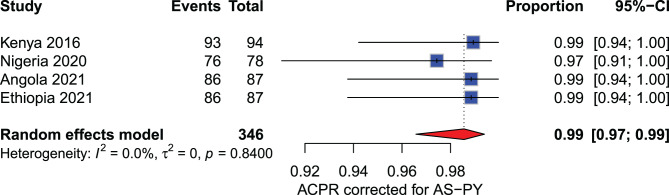
Forest plot for artesunate-pyronaridine (AS-PY) PCR adjusted based on the per-protocol analysis in sub-Saharan Africa from 2010 to 2024

**Artesunate - sulfadoxine pyrimethamine (AS+SP):** From 2010 to 2016, 1,595 participants were included in the meta-analysis. Before 2014, the reported PCR-corrected ACPR rates at D28 ranged from 91% to 100%. However, the PCR-corrected ACPR declined for studies conducted between 2015 and 2016, ranging from 86% to 90% (Fig. 8, see Supplementary Figure 2, Additional File 2).

**Artesunate - mefloquine (AS-MQ):** The only study included was conducted in Senegal in 2010, which enrolled 151 participants and reported a PCR-corrected ACPR rate at D28 of 99% (150/151) and at D42 of 98.5% (69/70).

### Temporal trend of act efficacy per country in different African regions

**Fig. 9:**
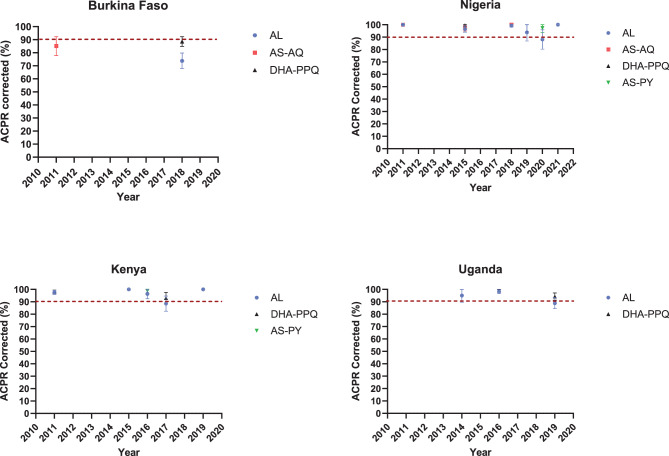
Temporal trend of ACPR-PCR adjusted per country from 2010 to 2024

**West Africa:** The ACPR corrected (≥90%) for ACT drugs has generally remained high across most of countries in West Africa over time. However, some countries reported a decline in the efficacy of AL (Fig. 9 and See Supplementary Figure 3, Additional File 2). In Nigeria, the temporal trends of PCR-corrected ACPR for AL showed a reduction in efficacy, with a recent study conducted in 2020 reporting a PCR-corrected ACPR of 88.10% (59/67). Conversely, another report in 2021 demonstrated a perfect PCR-corrected ACPR rate of 100%. The PCR-corrected ACPR for AS-AQ, DHA-PPQ, and AS-PY consistently exceeded 90%. Notably, a study conducted in 2018 reported a PCR-corrected ACPR of 100% for AS-AQ, while a study conducted in 2020 reported a PCR-corrected ACPR of 97.40% (76/78; 95% CI 93.87–100%) for AS-PY. In Burkina Faso, all studies on AS-AQ (2011), AL (2018), and DHA-PPQ (2018) reported PCR-corrected ACPR rates below 90%, with values of 85.10% (80/94; 95% CI 77.90–92.30%), 73.80% (155/210; 95% CI 67.85–79.75%), and 88.61% (249/281; 95% CI 84.90–92.32%), respectively. In Mali, the temporal trends of PCR-corrected ACPR for AL remained higher than 90% over time (range between 90 and 100%), with a slight decline in the efficacy of AL in 2016, with a PCR-corrected rate of 90.95% (181/199; 95% CI 86.96–94.94).

**Central Africa:** The PCR-corrected ACPR over time for AL, AS-AQ, and DHA-PPQ remained above 90% in most Central African countries. However, for AL in Angola, the PCR-corrected ACPR confidence interval showed a lower limit inferior to 90% with a rate of 85.80% (94/103; 95% CI: 85.80%-96.71%), 89.00% (146/157; 95% CI: 89.00%-96.98%), 87.49% (163/178; 95% CI: 87.49%-95.65%), and 86.81% (162/178; 95% CI: 86.81%-95.21%) in 2012, 2013, 2015, and 2021 respectively. A similar trend was observed for AS-AQ, with PCR-corrected ACPR with a lower limit of 95% CI at 88.41% in Gabon in 2018 (40/42; 95% CI : 88.41–100%) and 87.85% in the Republic of Congo in 2017 (49/52; 95% CI : 87.85–100%). (See Supplementary Figure 4, Additional File 2)

**East Africa:** The PCR-corrected ACPR for ACT drugs was generally high across most countries, consistently exceeding 90% over time. However, variations were noted in Kenya and Uganda. In Kenya, the efficacy of AL dropped below 90% in 2017, and accounted for 88.50% (95% CI: 82.37%–94.63%). Similarly, in Uganda, from 2014 to 2019, the PCR-corrected ACPR consistently remained above 90%, but dropped in 2019 to a rate of 88.89% (95% CI: 84.51–93.27%) (Fig. 9 and See Supplementary Figure 5, Additional File 2).

## Discussion

The efficacy of AS-AQ and DHA-PPQ remained consistently high, exceeding 90% across African countries. However, a decline in AL efficacy below 90% was observed in some countries after 2015, including Burkina Faso, Nigeria, Uganda, Angola, and Kenya. Although limited studies have examined AS-PY, its efficacy remained high at approximately 95%.

Our findings indicate that AS-AQ, DHA-PPQ, and AS-PY remain efficacious, with PCR-corrected efficacies being over the WHO-recommended 90% threshold for maintaining current treatment policies [1, 24]. These ACTs have demonstrated sustained efficacy in treating uncomplicated *P. falciparum* malaria for over a decade, however, evidence for AS–PY remains scarce due to the small number of available studies. These results align with Marwa et al.‘s meta-analysis [40]. The studies conducted before 2010 reported high ACT efficacy, with treatment failure rates generally below 5% [41, 42]. This observation aligns with our findings, which demonstrated a PCR-corrected ACPR approaching 100% between 2010 and 2015 in the present meta-analysis. Collectively, the data show a progressive decline in ACT efficacy after 2010, with a more pronounced increase in treatment failures observed beyond 2015. Unlike *P. falciparum* populations in Southeast Asia, those in Africa appear to experience lower evolutionary pressure for the rapid development of ACT resistance, likely due to differences in transmission intensity, historical drug use patterns, and parasite population structure [5, 43–47]. In Southeast Asia, the widespread use of artemisinin monotherapy before ACT adoption and the antimalarial mass drug administration in large populations likely contributed to the emergence of resistance [48–50]. In contrast, high malaria endemicity in Africa leads to frequent parasite exposure and the development of naturally acquired immunity. Individuals with premunition in malaria often experience asymptomatic infections and are less likely to seek treatment, thereby reducing the selective pressure for resistance. Additionally, the lower prevalence of background mutations (ferredoxin (*fd*), multidrug resistance 2 (*mdr2*), apicoplast ribosomal protein S10 (*arps10*)) in African *P. falciparum* strains compared to those in Southeast Asia further supports the sustained efficacy of ACTs in Africa [17, 51–55].

The decline in AL efficacy observed since 2015 is a growing concern, particularly in West Africa (Nigeria, Burkina Faso) and East Africa (Uganda, Kenya), where AL remains a key first-line malaria treatment. These countries significantly contribute to Africa’s malaria burden, with Nigeria, Uganda, and Burkina Faso accounting for 25.9%, 4.8%, and 3.1% of global malaria cases, respectively, in 2023 [1]. The widespread use of AL for uncomplicated malaria in these high-burden regions may exert substantial selective pressure on *P. falciparum*, potentially accelerating the development of resistance. Although AL efficacy has declined in both regions, East Africa faces an additional challenge: the emergence and rising prevalence of *PfKelch13* mutations [5, 16–19], whereas such validated mutations have not yet been reported in West Africa. In addition, the decrease in AL efficacy in East Africa was associated with the emergence of *PfKelch13* mutations in this region, suggesting a connection between these mutations and artemisinin partial resistance. To overcome this threat, targeted surveillance and proactive intervention strategies (multiple first-line therapies (MFT), rotation of ACT regimens, ensuring strict adherence to treatment guidelines, and promoting correct dosing and supervision of treatments) are urgently needed to preserve ACT efficacy in sub-Saharan Africa.

The WHO recommends the Multiple First-Line Therapies (MFT) strategy as one of the alternatives to address antimalarial drug resistance. The strategy aims to improve resistance detection, ensure timely responses, and minimise the impact of drug resistance. A modelling study showed that compared with a single first-line therapy, multiple first-line therapies significantly delay resistance emergence and treatment failure, and slow the spread of resistance once resistance emerges [56, 57]. Indeed, this approach limits the emergence of resistance by exposing the parasite population to multiple antimalarial agents. MFT involves the concurrent or rotational use of two or more effective ACTs to treat uncomplicated malaria [57]. Based on our meta-analysis, AL, AS-AQ, DHA-PPQ, and AS-PY are strong candidates for implementation within this strategy. The high efficacy of AS-PY suggests it could serve as an alternative to AL in regions where AL efficacy is declining.

In Burkina Faso, studies on ACT efficacy trends have reported declines below 90% for AS-AQ, AL, and DHA-PPQ [22, 58]. However, methodological inconsistencies were identified in these studies, particularly the misclassification of re-infections as recrudescence instead of excluding them, due to the PCR correction methods, which may have led to an underestimation of efficacy [59]. The WHO recommends using three genetic markers (*msp1*, *msp2*, and *glurp*) for PCR correction, as using fewer markers may lower the estimated efficacy since recrudescence is only confirmed when at least one allele at each locus matches between baseline and failure samples [24, 59]. For instance, the study by Gansané et al. [22], which reported inadequate efficacy of AL and DHA-PPQ, may have been impacted by some limitations. The researchers reported that children in the 5–9 kg weight group received half a tablet of AL instead of the recommended whole tablet, due to an error in the dosing table of the protocol approved by the ethics committee and regulatory authority. Despite this dosing error, sensitivity analyses excluding this weight group demonstrated that the overall findings remained consistent. Moreover, fatty food—recommended by the manufacturer to enhance lumefantrine absorption—was not systematically administered to children in the AL group [22]. Consequently, the absence of pharmacokinetic data on lumefantrine limited the ability to assess adequate drug exposure and absorption. Additionally, the high rate of glurp amplification failure raises concerns about the reliability of some PCR-corrected efficacy estimates [59]. The use of more sensitive genotyping markers, such as microsatellites instead of glurp, could improve the accuracy and robustness of molecular correction analyses. Moreover, implementing Next Generation Sequencing (NGS) would be ideal, though it first requires strengthening local capacity and implies higher costs. [59–62].

Burkina Faso revised its treatment policy in 2017, replacing AS-AQ with DHA-PPQ as the first-line therapy because SMC with SP-AQ is used nationwide. In 2021, the country introduced AS-PY and implemented the MFT strategy as a pilot in a few districts [63, 64]. An updated Therapeutic Efficacy Study (TES), conducted in collaboration with the National Malaria Control Programme (NMCP), is essential to verify and confirm the observed trends in the decline of AL and DHA-PPQ efficacy, in line with WHO recommendations. Despite observed declines in ACT efficacy in Burkina Faso, no *PfKelch13* mutations associated with partial artemisinin resistance have been reported, and there is no evidence of multidrug resistance to amodiaquine, lumefantrine, or piperaquine [7, 22, 65, 66]. However, continuous surveillance is crucial for detecting and promptly responding to emerging resistance.

The temporal trends in ACT efficacy in Nigeria, Uganda, and Kenya indicate that AL efficacy dropped below 90% in 2020 [67], 2019 [23], and 2017 [68], respectively. However, later studies reported AL efficacy at 100% in Nigeria (2021) [69] and Kenya (2019) [70], suggesting that the reductions observed in earlier studies may have been context-specific or influenced by local factors. These findings indicate that more recent data do not confirm the previously reported declines in AL efficacy, highlighting the importance of ongoing surveillance to monitor treatment performance across different settings accurately. Despite this, the independent emergence and spread of the *PfKelch13* R561H mutation in Uganda and Rwanda, associated with partial artemisinin resistance [5], is a significant concern, and urgent measures are necessary to mitigate the spread of resistance to the whole continent.

AS+SP was the main antimalarial treatment used in Sudan and Somalia [2] until its efficacy declined between 2015 and 2016, as shown in our meta-analysis [71, 72]. Although no *PfKelch13* mutations linked to artemisinin partial resistance have been reported until 2016 in these countries [73], widespread resistance to sulfadoxine-pyrimethamine (SP), the partner drug, likely contributed to the observed reduction in treatment efficacy. In Somalia, SP resistance emerged between 2011 and 2015, shortly after its introduction, necessitating a shift to AL. In 2016–2017, AS+SP was replaced with AL as the first-line treatment and DHA-PPQ as the second-line treatment due to a SP treatment failure rate exceeding 10% [71, 74, 75]. Warsame et al. reported a PCR-corrected treatment failure rate of 12.3% in patients treated with AS+SP, with all failures occurring in individuals carrying the quintuple mutation (51I/108N + 437 G/540E/581 G), highlighting extensive SP resistance [74]. Subsequent studies conducted between 2017 and 2020 confirmed the high efficacy of AL and DHA-PPQ, supporting their continued use in Somalia [76].

A similar pattern was observed in Sudan, where a high prevalence of SP resistance-associated quintuple mutations was reported [77, 78]. In response, Sudan revised its treatment guidelines, to introduce AL and DHA-PPQ as first- and second-line therapies. Studies conducted between 2017 and 2020 have consistently demonstrated the sustained efficacy of these therapies [79].

This systematic review analyzes data at the national level, which limits the ability to account for variations in malaria transmission intensity within countries or specific sites, which may influence the efficacy of ACTs. Additionally, some studies did not strictly adhere to the WHO TES guidelines, potentially affecting reported efficacy estimates. Evaluating the impact of methodological inconsistencies on these findings is crucial.

## Conclusion

This review supports the sustained high efficacy of AS-AQ, DHA-PPQ, and AS-PY, with treatment success rates exceeding the WHO threshold, indicating no immediate need for a policy change in sub-Saharan Africa. While AL has generally maintained good efficacy over time, few countries have reported a decline. AS-PY is a promising option for first-line malaria treatment to counter the declining efficacy of AL. Although few countries have already incorporated AS-PY into their first-line regimens, wider adoption will require careful assessment of operational feasibility and implementation considerations. Continuous therapeutic efficacy monitoring in collaboration with National Malaria Control Programs remains essential to prevent the emergence and spread of ACT resistance.

## Electronic supplementary material

Below is the link to the electronic supplementary material.

Supplementary Material 1

Supplementary Material 2

## Data Availability

No datasets were generated or analysed during the current study.

## Abbreviations

ACT: Artemisinin-based combination therapies
AL: Artemether–lumefantrine
PCR: Polymerase chain reaction
AS-PY: Artesunate-pyronaridine
DHA-PPQ: Dihydroartemisinin-piperaquine
AS-MQ: Artesunate-mefloquine
AS+SP: Artesunate+sulfadoxine-primethamine
AS-AQ: Artesunate-amodiaquine
ACPR: Adequate clinical and parasitological response
PRISMA: Preferred reporting items for systematic review and meta-analysis

## References

[1] World malaria report 2024 n.d. Accessed March 12, 2025. https://www.who.int/teams/global-malaria-programme/reports/world-malaria-report-2024.

[2] Artemisinin resistance and artemisinin-based combination therapy efficacy (December 2019) n.d.

[3] Noedl H, Se Y, Schaecher K, Smith BL, Socheat D, Fukuda MM, et al. Evidence of artemisinin-resistant malaria in western Cambodia. N Engl J Med. 2008;359:2619–20. 10.1056/NEJMc0805011. 19064625 10.1056/NEJMc0805011

[4] Dondorp AM, Nosten F, Yi P, Das D, Phyo AP, Tarning J, et al. Artemisinin resistance in Plasmodium falciparum malaria. N Engl J Med. 2009;361:455–67. 10.1056/NEJMoa0808859. 19641202 10.1056/NEJMoa0808859 PMC3495232

[5] Rosenthal PJ, Asua V, Bailey JA, Conrad MD, Ishengoma DS, Kamya MR, et al. The emergence of artemisinin partial resistance in Africa: how do we respond? Lancet Infect Dis. 2024;24:e591–600. 10.1016/S1473-3099(24)00141-5. 38552654 10.1016/S1473-3099(24)00141-5 PMC12954456

[6] Kamau E, Campino S, Amenga-Etego L, Drury E, Ishengoma D, Johnson K, et al. K13-propeller polymorphisms in Plasmodium falciparum parasites from Sub-Saharan Africa. J Infect Dis. 2015;211:1352–55. 25367300 10.1093/infdis/jiu608 PMC4827505

[7] Ménard D, Khim N, Beghain J, Adegnika AA, Shafiul-Alam M, Amodu O, et al. A worldwide map of plasmodium falciparum K13-propeller polymorphisms. N Engl J Med. 2016;374:2453–64. 10.1056/NEJMoa1513137. 27332904 10.1056/NEJMoa1513137 PMC4955562

[8] Ndwiga L, Kimenyi KM, Wamae K, Osoti V, Akinyi M, Omedo I, et al. A review of the frequencies of Plasmodium falciparum Kelch 13 artemisinin resistance mutations in Africa. Int J Parasitol Drugs Drug Resist. 2021;16:155–61. 10.1016/j.ijpddr.2021.06.001. 34146993 10.1016/j.ijpddr.2021.06.001 PMC8219943

[9] Rosenthal PJ, Asua V, Conrad MD. Emergence, transmission dynamics and mechanisms of artemisinin partial resistance in malaria parasites in Africa. Nat Rev Microbiol. 2024;22:373–84. 10.1038/s41579-024-01008-2. 38321292 10.1038/s41579-024-01008-2

[10] Uwimana A, Umulisa N, Venkatesan M, Svigel SS, Zhou Z, Munyaneza T, et al. Association of plasmodium falciparum kelch13 R561H genotypes with delayed parasite clearance in Rwanda: an open-label, single-arm, multicentre, therapeutic efficacy study. Lancet Infect Dis. 2021;21:1120–28. 10.1016/S1473-3099(21)00142-0. 33864801 10.1016/S1473-3099(21)00142-0 PMC10202849

[11] Mesia Kahunu G, Wellmann Thomsen S, Wellmann Thomsen L, Muhindo Mavoko H, Mitashi Mulopo P, Filtenborg Hocke E, et al. Identification of the PfK13 mutations R561H and P441L in the democratic Republic of Congo. Int J Infect Dis IJID Off Publ Int Soc Infect Dis. 2024;139:41–49. 10.1016/j.ijid.2023.11.026. 10.1016/j.ijid.2023.11.026 38016502

[12] Abebe W, Mekuanint A, Asmare Z, Woldesenbet D, Mihret Y, Setegn A, et al. Prevalence of molecular markers of chloroquine resistance in malaria parasites in East Africa: a systematic review and meta-analysis. J Glob Antimicrob Resist. 2024. 10.1016/j.jgar.2024.12.019. 39725320 10.1016/j.jgar.2024.12.019

[13] Wang S, Huang F, Yan H, Yin J, Xia Z. A review of malaria molecular markers for drug resistance in Plasmodium falciparum and Plasmodium vivax in China. Front Cell Infect Microbiol. 2023;13:1167220. 10.3389/fcimb.2023.1167220. 37228664 10.3389/fcimb.2023.1167220 PMC10203619

[14] Assefa A, Fola AA, Tasew G. Emergence of plasmodium falciparum strains with artemisinin partial resistance in East Africa and the Horn of Africa: is there a need to panic? Malar J. 2024;23:34. 10.1186/s12936-024-04848-8. 38273360 10.1186/s12936-024-04848-8 PMC10809756

[15] Moser KA, Madebe RA, Aydemir O, Chiduo MG, Mandara CI, Rumisha SF, et al. Describing the current status of Plasmodium falciparum population structure and drug resistance within mainland Tanzania using molecular inversion probes. Mol Ecol. 2021;30:100–13. 10.1111/mec.15706. 33107096 10.1111/mec.15706 PMC8088766

[16] Uwimana A, Legrand E, Stokes BH, Ndikumana J-L, Warsame M, Umulisa N, et al. Emergence and clonal expansion of in vitro artemisinin-resistant plasmodium falciparum kelch13 R561H mutant parasites in Rwanda. Nat Med. 2020;26:1602–08. 10.1038/s41591-020-1005-2. 32747827 10.1038/s41591-020-1005-2 PMC7541349

[17] Conrad MD, Asua V, Garg S, Giesbrecht D, Niaré K, Smith S, et al. Evolution of partial resistance to artemisinins in malaria parasites in Uganda. N Engl J Med. 2023;389:722–32. 10.1056/NEJMoa2211803. 37611122 10.1056/NEJMoa2211803 PMC10513755

[18] Fola AA, Feleke SM, Mohammed H, Brhane BG, Hennelly CM, Assefa A, et al. Plasmodium falciparum resistant to artemisinin and diagnostics have emerged in Ethiopia. Nat Microbiol. 2023;8:1911–19. 10.1038/s41564-023-01461-4. 37640962 10.1038/s41564-023-01461-4 PMC10522486

[19] Mihreteab S, Platon L, Berhane A, Stokes BH, Warsame M, Campagne P, et al. Increasing prevalence of artemisinin-resistant HRP2-negative malaria in Eritrea. N Engl J Med. 2023;389:1191–202. 10.1056/NEJMoa2210956. 37754284 10.1056/NEJMoa2210956 PMC10539021

[20] Dimbu PR, Horth R, Cândido ALM, Ferreira CM, Caquece F, Garcia LEA, et al. Continued Low Efficacy of Artemether-Lumefantrine in Angola in 2019. Antimicrob Agents Chemother. 2021;65:e01949–20. 10.1128/AAC.01949-20. 33168604 10.1128/AAC.01949-20 PMC7849008

[21] Moriarty LF, Nkoli PM, Likwela JL, Mulopo PM, Sompwe EM, Rika JM, et al. Therapeutic efficacy of artemisinin-based combination therapies in Democratic Republic of the Congo and investigation of molecular markers of antimalarial resistance. Am J Trop Med Hyg. 2021;105:1067–75. 10.4269/ajtmh.21-0214. 34491220 10.4269/ajtmh.21-0214 PMC8592145

[22] Gansané A, Moriarty LF, Ménard D, Yerbanga I, Ouedraogo E, Sondo P, et al. Anti-malarial efficacy and resistance monitoring of artemether-lumefantrine and dihydroartemisinin-piperaquine shows inadequate efficacy in children in Burkina Faso, 2017–2018. Malar J. 2021;20:48. 10.1186/s12936-021-03585-6. 33468147 10.1186/s12936-021-03585-6 PMC7816451

[23] Ebong C, Sserwanga A, Namuganga JF, Kapisi J, Mpimbaza A, Gonahasa S, et al. Efficacy and safety of artemether-lumefantrine and dihydroartemisinin-piperaquine for the treatment of uncomplicated plasmodium falciparum malaria and prevalence of molecular markers associated with artemisinin and partner drug resistance in Uganda. Malar J. 2021;20:484. 10.1186/s12936-021-04021-5. 34952573 10.1186/s12936-021-04021-5 PMC8709966

[24] Report on antimalarial drug efficacy, resistance and response: 10 years of surveillance (2010-2019) n.d. accessed March 15, 2023. https://www.who.int/publications-detail-redirect/9789240012813.

[25] Borrmann S, Sasi P, Mwai L, Bashraheil M, Abdallah A, Muriithi S, et al. Declining responsiveness of plasmodium falciparum infections to artemisinin-based combination treatments on the Kenyan coast. PLoS One. 2011;6:e26005. 10.1371/journal.pone.0026005. 22102856 10.1371/journal.pone.0026005 PMC3213089

[26] Hawkes M, Conroy AL, Opoka RO, Namasopo S, Zhong K, Liles WC, et al. Slow clearance of plasmodium falciparum in severe pediatric malaria, Uganda, 2011-2013. Emerg Infect Dis. 2015;21:1237–39. 10.3201/eid2107.150213. 26079933 10.3201/eid2107.150213 PMC4480400

[27] Kiaco K, Teixeira J, Machado M, Do Rosário V, Lopes D. Evaluation of artemether-lumefantrine efficacy in the treatment of uncomplicated malaria and its association with pfmdr1, pfatpase6 and K13-propeller polymorphisms in Luanda, Angola. Malar J. 2015;14:504. 10.1186/s12936-015-1018-3. 26670642 10.1186/s12936-015-1018-3 PMC4681156

[28] Rathmes G, Rumisha SF, Lucas TCD, Twohig KA, Python A, Nguyen M, et al. Global estimation of anti-malarial drug effectiveness for the treatment of uncomplicated plasmodium falciparum malaria 1991–2019. Malar J. 2020;19:374. 10.1186/s12936-020-03446-8. 33081784 10.1186/s12936-020-03446-8 PMC7573874

[29] Mathenge PG, Low SK, Vuong NL, Mohamed MYF, Faraj HA, Alieldin GI, et al. Efficacy and resistance of different artemisinin-based combination therapies: a systematic review and network meta-analysis. Parasitol Int. 2020;74:101919. 10.1016/j.parint.2019.04.016. 31015034 10.1016/j.parint.2019.04.016

[30] Derbie A, Mekonnen D, Adugna M, Yeshitela B, Woldeamanuel Y, Abebe T. Therapeutic efficacy of artemether-lumefantrine (Coartem®) for the treatment of uncomplicated falciparum malaria in Africa: a systematic review. J Parasitol Res. 2020;2020:7371681. 10.1155/2020/7371681. 33145101 10.1155/2020/7371681 PMC7599419

[31] Shibeshi W, Alemkere G, Mulu A, Engidawork E. Efficacy and safety of artemisinin-based combination therapies for the treatment of uncomplicated malaria in pediatrics: a systematic review and meta-analysis. BMC Infect Dis. 2021;21:326. 10.1186/s12879-021-06018-6. 33827422 10.1186/s12879-021-06018-6 PMC8028735

[32] Whegang Youdom S, Chiabi A, Basco LK. Monitoring the efficacy and safety of artemisinin-based combination therapies: a review and network meta-analysis of antimalarial therapeutic efficacy trials in Cameroon. Drugs RD. 2019;19:1–14. 10.1007/s40268-018-0259-3. 10.1007/s40268-018-0259-3 PMC6380963 30656608

[33] Mb A. Therapeutic efficacy of artemether-lumefantrine in the treatment of uncomplicated plasmodium falciparum malaria in Ethiopia: a systematic review and meta-analysis. Infect Dis Poverty. 2017, 6. 10.1186/s40249-017-0372-5. 10.1186/s40249-017-0372-5 PMC5686809 29137664

[34] Bello SO, Chika A, Abdulgafar JO. Artesunate plus amodiaquine (AS+AQ) versus artemether -lumefantrine (AL) for the treatment of uncomplicated plasmodium falciparum malaria in sub-Saharan Africa-a meta-analysis. Afr J Infect Dis. 2010;4:20–28. 10.4314/ajid.v4i2.55149. 23878697 10.4314/ajid.v4i2.55149 PMC3497848

[35] Moher D, Shamseer L, Clarke M, Ghersi D, Liberati A, Petticrew M, et al. Preferred reporting items for systematic review and meta-analysis protocols (PRISMA-P) 2015 statement. Syst Rev. 2015;4:1. 10.1186/2046-4053-4-1. 25554246 10.1186/2046-4053-4-1 PMC4320440

[36] Ouzzani M, Hammady H, Fedorowicz Z, Elmagarmid A. Rayyan—a web and mobile app for systematic reviews. Syst Rev. 2016;5:210. 10.1186/s13643-016-0384-4. 27919275 10.1186/s13643-016-0384-4 PMC5139140

[37] ACT Efficacy Africa. n.d. Accessed March 13, 2025. https://app.covidence.org/reviews/359664.

[38] Study Quality Assessment Tools | NHLBI, NIH n.d. Accessed March 13, 2025. https://www.nhlbi.nih.gov/health-topics/study-quality-assessment-tools.

[39] Sedgwick P. Meta-analyses: heterogeneity and subgroup analysis. BMJ. 2013;346:f4040. 10.1136/bmj.f4040.

[40] Marwa K, Kapesa A, Baraka V, Konje E, Kidenya B, Mukonzo J, et al. Therapeutic efficacy of artemether-lumefantrine, artesunate-amodiaquine and dihydroartemisinin-piperaquine in the treatment of uncomplicated plasmodium falciparum malaria in Sub-Saharan Africa: a systematic review and meta-analysis. PLoS One. 2022;17:e0264339. 10.1371/journal.pone.0264339. 35271592 10.1371/journal.pone.0264339 PMC8912261

[41] Zwang J, Olliaro P, Barennes H, Bonnet M, Brasseur P, Bukirwa H, et al. Efficacy of artesunate-amodiaquine for treating uncomplicated falciparum malaria in sub-Saharan Africa: a multi-centre analysis. Malar J. 2009;8:203. 10.1186/1475-2875-8-203. 19698172 10.1186/1475-2875-8-203 PMC2745424

[42] Ogbonna A, Uneke CJ. Artemisinin-based combination therapy for uncomplicated malaria in sub-Saharan Africa: the efficacy, safety, resistance and policy implementation since Abuja 2000. Trans R Soc Trop Med Hyg. 2008;102:621–27. 10.1016/j.trstmh.2008.03.024. 18499204 10.1016/j.trstmh.2008.03.024

[43] Woodrow CJ, White NJ. The clinical impact of artemisinin resistance in Southeast Asia and the potential for future spread. FEMS Microbiol Rev. 2017;41:34–48. 10.1093/femsre/fuw037. 27613271 10.1093/femsre/fuw037 PMC5424521

[44] Masserey T, Lee T, Golumbeanu M, Shattock AJ, Kelly SL, Hastings IM, et al. The influence of biological, epidemiological, and treatment factors on the establishment and spread of drug-resistant plasmodium falciparum. eLife. 2022;11:e77634. 10.7554/eLife.77634. 35796430 10.7554/eLife.77634 PMC9262398

[45] Menard D, Dondorp A. Antimalarial drug resistance: a threat to malaria elimination. Cold Spring Harb Perspect Med. 2017;7:a025619. 10.1101/cshperspect.a025619. 28289248 10.1101/cshperspect.a025619 PMC5495053

[46] Balmer AJ, White NF, Ünlü ES, Lee C, Pearson RD, Almagro-Garcia J, et al. Understanding the global rise of artemisinin resistance: insights from over 100,000 plasmodium falciparum samples. eLife. n.d;14:e105544. 10.7554/eLife.105544. 10.7554/eLife.105544 PMC12490857 41037007

[47] Balikagala B, Fukuda N, Ikeda M, Katuro OT, Tachibana S-I, Yamauchi M, et al. Evidence of artemisinin-resistant malaria in Africa. N Engl J Med. 2021;385:1163–71. 10.1056/NEJMoa2101746. 34551228 10.1056/NEJMoa2101746

[48] Ishengoma DS, Mandara CI, Francis F, Talundzic E, Lucchi NW, Ngasala B, et al. Efficacy and safety of artemether-lumefantrine for the treatment of uncomplicated malaria and prevalence of Pfk13 and Pfmdr1 polymorphisms after a decade of using artemisinin-based combination therapy in mainland Tanzania. Malar J. 2019;18:88. 10.1186/s12936-019-2730-1. 30898164 10.1186/s12936-019-2730-1 PMC6427902

[49] Maude RJ, Socheat D, Nguon C, Saroth P, Dara P, Li G, et al. Optimising strategies for plasmodium falciparum malaria elimination in Cambodia: primaquine, mass drug administration and artemisinin resistance. PLoS One. 2012;7:e37166. 10.1371/journal.pone.0037166. 22662135 10.1371/journal.pone.0037166 PMC3360685

[50] Nguyen TD, Tran T-A, Parker DM, White NJ, Boni MF. Antimalarial mass drug administration in large populations and the evolution of drug resistance. PLoS Glob Public Health. 2023;3:e0002200. 10.1371/journal.pgph.0002200. 37494337 10.1371/journal.pgph.0002200 PMC10370688

[51] Ocan M, Ashaba FK, Mwesigwa S, Edgar K, Kamya MR, Nsobya SL. Prevalence of arps10, fd, pfmdr-2, pfcrt and pfkelch13 gene mutations in Plasmodium falciparum parasite population in Uganda. PLoS One. 2022;17:e0268095. 10.1371/journal.pone.0268095. 35511795 10.1371/journal.pone.0268095 PMC9070901

[52] Stokes BH, Dhingra SK, Rubiano K, Mok S, Straimer J, Gnädig NF, et al. Plasmodium falciparum K13 mutations in Africa and Asia impact artemisinin resistance and parasite fitness. eLife. 2021;10:e66277. 10.7554/eLife.66277. 34279219 10.7554/eLife.66277 PMC8321553

[53] Miotto O, Amato R, Ashley EA, MacInnis B, Almagro-Garcia J, Amaratunga C, et al. Genetic architecture of artemisinin-resistant plasmodium falciparum. Nat Genet. 2015;47:226–34. 10.1038/ng.3189. 25599401 10.1038/ng.3189 PMC4545236

[54] Kampoun T, Srichairatanakool S, Prommana P, Shaw PJ, Green JL, Knuepfer E, et al. Apicoplast ribosomal protein S10-V127M enhances artemisinin resistance of a Kelch13 transgenic plasmodium falciparum. Malar J. 2022;21:302. 10.1186/s12936-022-04330-3. 36303209 10.1186/s12936-022-04330-3 PMC9615251

[55] Balikagala B, Mita T, Ikeda M, Sakurai M, Yatsushiro S, Takahashi N, et al. Absence of in vivo selection for K13 mutations after artemether–lumefantrine treatment in Uganda. Malar J. 2017;16:23. 10.1186/s12936-016-1663-1. 28068997 10.1186/s12936-016-1663-1 PMC5223472

[56] Boni MF, Smith DL, Laxminarayan R. Benefits of using multiple first-line therapies against malaria. Proc Natl Acad Sci U S A. 2008;105:14216–21. 10.1073/pnas.0804628105. 18780786 10.1073/pnas.0804628105 PMC2544604

[57] Multiple first-line therapies as part of the response to antimalarial drug resistance. n.d. https://www.who.int/publications/i/item/9789240103603. accessed March 10, 2025.

[58] Coulibaly B, Pritsch M, Bountogo M, Meissner PE, Nebié E, Klose C, et al. Efficacy and safety of triple combination therapy with artesunate-amodiaquine–methylene blue for falciparum malaria in children: a randomized controlled trial in Burkina Faso. J Infect Dis. 2015;211:689–97. 10.1093/infdis/jiu540. 25267980 10.1093/infdis/jiu540

[59] Rasmussen C, Ringwald P. Is there evidence of anti-malarial multidrug resistance in Burkina Faso? Malar J. 2021;20:320. 10.1186/s12936-021-03845-5. 34281562 10.1186/s12936-021-03845-5 PMC8287766

[60] Kamaliddin C, Joste V, Hubert V, Kendjo E, Argy N, Houze S. Evaluation of pcr to monitor plasmodium falciparum treatment efficacy in a nonendemicity setting. J Clin Microbiol. 2019;58:e01080–19. 10.1128/JCM.01080-19. 31666363 10.1128/JCM.01080-19 PMC6935925

[61] Methods and techniques for clinical trials on antimalarial drug efficacy: genotyping to identify parasite populations. n.d. https://www.who.int/publications/i/item/9789241596305. accessed March 14, 2025.

[62] Felger I, Snounou G, Hastings I, Moehrle JJ, Beck H-P. Pcr correction strategies for malaria drug trials: updates and clarifications. Lancet Infect Dis. 2020;20:e20–5. 10.1016/S1473-3099(19)30426-8. 31540841 10.1016/S1473-3099(19)30426-8

[63] Kaboré JMT, Siribié M, Hien D, Soulama I, Barry N, Baguiya A, et al. Feasibility and acceptability of a strategy deploying multiple first-line artemisinin-based combination therapies for uncomplicated malaria in the health district of Kaya, Burkina Faso. Trop Med Infect Dis. 2023;8:195. 10.3390/tropicalmed8040195. 37104321 10.3390/tropicalmed8040195 PMC10145444

[64] Audibert C, Aspinall A, Tchouatieu A-M, Hugo P. Evaluation of segmentation, rotation, and geographic delivery approaches for deployment of multiple first-line treatment (MFT) to respond to antimalarial drug resistance in Africa: a qualitative study in seven sub-sahara countries. Trop Med Infect Dis. 2024;9:93. 10.3390/tropicalmed9050093. 38787026 10.3390/tropicalmed9050093 PMC11125622

[65] Leroy D, Macintyre F, Adoke Y, Ouoba S, Barry A, Mombo-Ngoma G, et al. African isolates show a high proportion of multiple copies of the plasmodium falciparum plasmepsin-2 gene, a piperaquine resistance marker. Malar J. 2019;18:126. 10.1186/s12936-019-2756-4. 30967148 10.1186/s12936-019-2756-4 PMC6457011

[66] Sondo P, Derra K, Diallo Nakanabo S, Tarnagda Z, Kazienga A, Zampa O, et al. Artesunate-amodiaquine and artemether-lumefantrine therapies and selection of Pfcrt and Pfmdr1 alleles in Nanoro, Burkina Faso. PLoS One. 2016;11:e0151565. 10.1371/journal.pone.0151565. 27031231 10.1371/journal.pone.0151565 PMC4816516

[67] Falade CO, Orimadegun AE, Olusola FI, Michael OS, Anjorin OE, Funwei RI, et al. Efficacy and safety of pyronaridine–artesunate versus artemether–lumefantrine in the treatment of acute uncomplicated malaria in children in South-West Nigeria: an open-labelled randomized controlled trial. Malar J. 2023;22:154. 10.1186/s12936-023-04574-7. 37179349 10.1186/s12936-023-04574-7 PMC10182553

[68] Westercamp N, Owidhi M, Otieno K, Chebore W, Buff AM, Desai M, et al. Efficacy of artemether-lumefantrine and dihydroartemisinin-piperaquine for the treatment of uncomplicated plasmodium falciparum malaria among children in Western Kenya, 2016 to 2017. Antimicrob Agents Chemother. 2022;66:e0020722. 10.1128/aac.00207-22. 36036611 10.1128/aac.00207-22 PMC9487560

[69] Oyebola KM, Ligali FC, Owoloye AJ, Aina OO, Alo YM, Erinwusi B, et al. Assessing the therapeutic efficacy of artemether-lumefantrine for uncomplicated malaria in Lagos, Nigeria: a comprehensive study on treatment response and resistance markers. Malar J. 2024;23:261. 10.1186/s12936-024-05088-6. 39210367 10.1186/s12936-024-05088-6 PMC11360866

[70] Hamaluba M, van der PR, Weya J, Njuguna P, Ngama M, Kalume P, et al. Arterolane–piperaquine–mefloquine versus arterolane–piperaquine and artemether–lumefantrine in the treatment of uncomplicated plasmodium falciparum malaria in Kenyan children: a single-centre, open-label, randomised, non-inferiority trial. Lancet Infect Dis. 2021;21:1395–406. 10.1016/S1473-3099(20)30929-4. 34111412 10.1016/S1473-3099(20)30929-4 PMC8461080

[71] Warsame M, Hassan AH, Hassan AM, Arale AM, Jibril AM, Mohamud SA, et al. Efficacy of artesunate + sulphadoxine/pyrimethamine and artemether + lumefantrine and dhfr and dhps mutations in Somalia: evidence for updating the malaria treatment policy. Trop Med Int Health TM IH. 2017;22:415–22. 10.1111/tmi.12847. 28151566 10.1111/tmi.12847

[72] Adeel AA, Elnour FAA, Elmardi KA, Abd-Elmajid MB, Elhelo MM, Ali MS, et al. High efficacy of artemether-lumefantrine and declining efficacy of artesunate + sulfadoxine-pyrimethamine against plasmodium falciparum in Sudan (2010–2015): evidence from in vivo and molecular marker studies. Malar J. 2016;15:285. 10.1186/s12936-016-1339-x. 27209063 10.1186/s12936-016-1339-x PMC4875683

[73] Strategy to respond to antimalarial drug resistance in Africa n.d. Accessed March 14, 2025. https://www.who.int/publications/i/item/9789240060265.

[74] Warsame M, Hassan AM, Barrette A, Jibril AM, Elmi HH, Arale AM, et al. Treatment of uncomplicated malaria with artesunate plus sulfadoxine–pyrimethamine is failing in Somalia: evidence from therapeutic efficacy studies and Pfdhfr and pfdhps mutant alleles. Trop Med Int Health. 2015;20:510–17. 10.1111/tmi.12458. 25583123 10.1111/tmi.12458

[75] Hassan SA, Mohamed Dirie A, Ahmed NR, Omar AI. Update on antimicrobial resistance in Somalia: current status, challenges, opportunities, and future perspectives. Heliyon. 2024;10:e39434. 10.1016/j.heliyon.2024.e39434. 39506942 10.1016/j.heliyon.2024.e39434 PMC11538744

[76] Warsame M, Hassan AM, Hassan AH, Jibril AM, Khim N, Arale AM, et al. High therapeutic efficacy of artemether-lumefantrine and dihydroartemisinin-piperaquine for the treatment of uncomplicated falciparum malaria in Somalia. Malar J. 2019;18:231. 10.1186/s12936-019-2864-1. 31296223 10.1186/s12936-019-2864-1 PMC6624891

[77] Mohamed NS, Abdelbagi H, Osman HA, Ahmed AE, Yousif AM, Edris YB, et al. A snapshot of plasmodium falciparum malaria drug resistance markers in Sudan: a pilot study. BMC Res Notes. 2020;13:512. 10.1186/s13104-020-05363-0. 33160417 10.1186/s13104-020-05363-0 PMC7648977

[78] Mohamed AO, Hussien M, Mohamed A, Suliman A, Elkando NS, Abdelbagi H, et al. Assessment of plasmodium falciparum drug resistance molecular markers from the Blue Nile State, Southeast Sudan. Malar J. 2020;19:78. 10.1186/s12936-020-03165-0. 32070355 10.1186/s12936-020-03165-0 PMC7029593

[79] Adam M, Nahzat S, Kakar Q, Assada M, Witkowski B, Tag Eldin Elshafie A, et al. Antimalarial drug efficacy and resistance in malaria-endemic countries in HANMAT-PIAM_net countries of the Eastern Mediterranean region 2016–2020: clinical and genetic studies. Trop Med Int Health n.d.;n/a. 10.1111/tmi.13929. 10.1111/tmi.13929 37705047

