# Supplementary material for: Spatio-temporal trends of artemisinin-based combination therapy efficacy from 2010 to 2024 in sub-Saharan Africa: a systematic review and meta-analysis
Source: BMC Infect Dis. 2025 Nov 25;25:1684. doi: 10.1186/s12879-025-12130-8 (PMC12670823; doi:10.1186/s12879-025-12130-8)

**Supplementary documen****ts**

**Supplementary Table 1: ACT tested in studies included in the systematic review**

| **ACT tested** | **Number of studies** | **Percentage (%)** |
| --- | --- | --- |
| AL | 42 | 33.3 |
| AL + AS-AQ | 32 | 25.4 |
| AL + DHA-PPQ | 16 | 12.7 |
| AL + AS-AQ + DHA-PPQ | 12 | 9.5 |
| AL + AS-AQ + DHA-PPQ + AS-PY | 01 | 0.8 |
| AL + AS-MQ | 01 | 0.8 |
| AL + AS+SP | 03 | 2.4 |
| AL + AS-PY | 02 | 1.6 |
| ASAQ | 09 | 7.1 |
| AS-AQ + DHA-PPQ | 01 | 0.8 |
| AS+SP | 02 | 1.6 |
| AS-PY | 01 | 0.8 |
| DHA-PPQ | 03 | 2.4 |
| DHA-PPQ + AS+SP | 01 | 0.8 |
| **Total** | **126** | **100** |

**AL:** Artemether Lumefantrine

**AS-AQ** : Artesunate Amodiaquine

**DHA-PPQ** : Dihydroartemisinin Piperaquine

**AS-MQ**: Artesunate Mefloquine

**AS+SP**: Artesunate Sulfadoxine Pyrimethamine

**AS-PY**: Artesunate Pyronaridine

**Supplementary Table 2: Distribution of ACT studies performed per country and year in sub-Saharan Africa. The number of studies conducted for each ACT is in the parentheses.**

| **Country** | **2010** | **2011** | **2012** | **2013** | **2014** | **2015** | **2016** | **2017** | **2018** | **2019** | **2020** | **2021** | **2022** | **2023** |
| --- | --- | --- | --- | --- | --- | --- | --- | --- | --- | --- | --- | --- | --- | --- |
| Angola |  |  | AL | AL, DHA-PPQ |  | AL, AS-AQ, DHA-PPQ |  | AL, AS-AQ, DHA-PPQ |  | AL, AS-AQ |  | AL, AS-AQ, DHA-PPQ, AS-PY |  |  |
| Benin |  |  |  |  | AL |  |  |  |  | AL |  |  |  |  |
| Burkina Faso |  | AS-AQ |  |  |  |  | AL, AS-AQ |  | AL, DHA-PPQ |  |  |  |  |  |
| Cameroon |  |  |  |  |  |  |  |  | DHA-PPQ |  | AL, AS-AQ |  |  |  |
| Central African Republic | AL, AS-AQ |  |  |  |  |  |  |  |  |  |  |  |  |  |
| Chad |  |  |  |  |  |  |  |  |  |  |  | AL, AS-AQ |  |  |
| Congo |  | AL, AS-AQ |  | AL, AS-AQ |  |  |  | AL, AS-AQ |  |  |  |  |  |  |
| Côte d'Ivoire |  |  | AL, AS-AQ |  |  |  | AL (4), AS-AQ (3) |  |  |  |  |  |  |  |
| Democratic Republic of the Congo |  |  | AL, AS-AQ, DHA-PPQ | AL | AL, AS-AQ (2) |  |  | AL (2), AS-AQ, DHA-PPQ |  |  |  |  |  |  |
| Equatorial Guinea |  |  |  |  |  |  |  |  | AL, AS-AQ |  |  |  |  |  |
| Eritrea |  |  |  |  |  | AS-AQ |  |  |  |  |  |  |  |  |
| Ethiopia |  | AL | AL | AL | AL | AL | AL | AL (2), DHA-PPQ |  |  |  | AL (2), AS-PY | AL | AL |
| Gabon |  |  |  |  |  | AL, AS-AQ |  |  | AL, AS-AQ |  |  |  |  |  |
| Gambia |  |  | AL | AL | AL |  |  |  |  |  |  |  |  |  |
| Ghana |  | AL | AS-AQ (2), DHA-PPQ |  | AL, AS-AQ (2) |  |  | AL, AS-AQ |  |  |  | DHA-PPQ |  |  |
| Guinea |  |  | AS-AQ |  |  |  | AL, AS-AQ |  |  |  |  |  |  |  |
| Guinea-Bissau |  |  |  |  |  | AL, DHA-PPQ |  |  |  |  |  |  |  |  |
| Kenya |  | AL (2), DHA-PPQ (2) |  |  |  | AL | AL, AS-PY | AL, DHA-PPQ |  | AL |  |  |  |  |
| Liberia |  |  |  |  |  |  |  |  | AL, AS-AQ |  |  |  |  |  |
| Malawi | AL |  |  |  | AL, AS-AQ |  |  |  |  |  |  |  |  |  |
| Mali |  |  | AL | AL (2), AS+SP | AL | AL, DHA-PPQ | AL, AS-AQ | AL | AL |  |  |  |  |  |
| Mauritania |  |  |  | AS-AQ |  |  |  |  |  |  |  |  |  |  |
| Mozambique |  |  | AL, AS-AQ |  |  | AL |  |  | AL, AS-AQ |  |  |  |  |  |
| Niger |  |  |  |  | AL, AS-AQ, DHA-PPQ |  |  | AL, AS-AQ |  |  | AL |  |  |  |
| Nigeria |  | AL, AS-AQ |  | AL |  | AL, AS-AQ, DHA-PPQ |  |  | AL, AS-AQ, DHA-PPQ | AL | AL, AS-PY | AL |  |  |
| Rwanda |  |  |  |  |  | AL, DHA-PPQ |  |  | AL |  |  |  |  |  |
| Senegal | AL, AS-MQ | AL | AL (2), AS-AQ, DHA-PPQ | AL | AL |  | AL, AS-AQ, DHA-PPQ | AL, AS-AQ, DHA-PPQ |  |  |  |  |  |  |
| Sierra Leone |  |  |  |  |  |  | AL, AS-AQ, DHA-PPQ |  |  |  |  |  |  |  |
| Somalia |  | AS+SP |  | AL |  | AL, AS+SP |  | AL, DHA-PPQ |  |  |  |  |  |  |
| Sudan | AL, AS+SP | AS+SP | AS+SP | AL, AS+SP | AL, AS+SP | AL, AS+SP | AS+SP (2), DHA-PPQ | AL, DHA-PPQ | AL |  | AL, DHA-PPQ |  |  |  |
| Togo |  |  |  | AL, AS-AQ |  |  |  | DHA-PPQ |  |  |  | AL, DHA-PPQ |  |  |
| Uganda |  |  | AL, DHA-PPQ |  | AL, AS-AQ |  | AL, DHA-PPQ |  |  | AL, DHA-PPQ |  |  |  |  |
| Tanzania |  | AL (3), AS-AQ | AL | AL (2) | AL (2) | AL (2), DHA-PPQ (2) | AL | AS-AQ, DHA-PPQ | AL | AL (2), DHA-PPQ | AS-AQ |  | AL (2), AS-AQ |  |
| Zambia |  |  | AL |  |  | AL |  |  |  |  |  |  |  |  |

**Supplementary Table 3: Number of studies included in the systematic review per country and year**

| **Country** | **2010** | **2011** | **2012** | **2013** | **2014** | **2015** | **2016** | **2017** | **2018** | **2019** | **2020** | **2021** | **2022** | **2023** | **Total** |
| --- | --- | --- | --- | --- | --- | --- | --- | --- | --- | --- | --- | --- | --- | --- | --- |
| Angola | 0 | 0 | 1 | 1 | 0 | 1 | 0 | 1 | 0 | 1 | 0 | 1 | 0 | 0 | 6 |
| Benin | 0 | 0 | 0 | 0 | 1 | 0 | 0 | 0 | 0 | 1 | 0 | 0 | 0 | 0 | 2 |
| Burkina Faso | 0 | 1 | 0 | 0 | 0 | 0 | 1 | 0 | 1 | 0 | 0 | 0 | 0 | 0 | 3 |
| Cameroon | 0 | 0 | 0 | 0 | 0 | 0 | 0 | 0 | 1 | 0 | 1 | 0 | 0 | 0 | 2 |
| Central African Republic | 1 | 0 | 0 | 0 | 0 | 0 | 0 | 0 | 0 | 0 | 0 | 0 | 0 | 0 | 1 |
| Chad | 0 | 0 | 0 | 0 | 0 | 0 | 0 | 0 | 0 | 0 | 0 | 1 | 0 | 0 | 1 |
| Congo | 0 | 1 | 0 | 1 | 0 | 0 | 0 | 1 | 0 | 0 | 0 | 0 | 0 | 0 | 3 |
| Côte d'Ivoire | 0 | 0 | 1 | 2 | 0 | 0 | 4 | 0 | 0 | 0 | 0 | 0 | 0 | 0 | 7 |
| Democratic Republic of Congo | 0 | 0 | 1 | 1 | 2 | 0 | 0 | 2 | 0 | 0 | 0 | 0 | 0 | 0 | 6 |
| Equatorial Guinea | 0 | 0 | 0 | 0 | 0 | 0 | 0 | 0 | 1 | 0 | 0 | 0 | 0 | 0 | 1 |
| Eritrea | 0 | 0 | 0 | 0 | 0 | 1 | 0 | 0 | 0 | 0 | 0 | 0 | 0 | 0 | 1 |
| Ethiopia | 0 | 1 | 1 | 1 | 1 | 1 | 1 | 2 | 0 | 0 | 0 | 3 | 1 | 1 | 13 |
| Gabon | 0 | 0 | 0 | 0 | 0 | 1 | 0 | 0 | 1 | 0 | 0 | 0 | 0 | 0 | 2 |
| Gambia | 0 | 0 | 1 | 1 | 1 | 0 | 0 | 0 | 0 | 0 | 0 | 0 | 0 | 0 | 3 |
| Ghana | 0 | 1 | 2 | 0 | 2 | 0 | 0 | 1 | 0 | 0 | 0 | 1 | 0 | 0 | 7 |
| Guinea | 0 | 0 | 1 | 0 | 0 | 0 | 1 | 0 | 0 | 0 | 0 | 0 | 0 | 0 | 2 |
| Guinea-Bissau | 0 | 0 | 0 | 0 | 0 | 1 | 0 | 0 | 0 | 0 | 0 | 0 | 0 | 0 | 1 |
| Kenya | 0 | 2 | 0 | 0 | 0 | 1 | 1 | 1 | 0 | 1 | 0 | 0 | 0 | 0 | 6 |
| Liberia | 0 | 0 | 0 | 0 | 0 | 0 | 0 | 0 | 1 | 0 | 0 | 0 | 0 | 0 | 1 |
| Malawi | 1 | 0 | 0 | 0 | 1 | 0 | 0 | 0 | 0 | 0 | 0 | 0 | 0 | 0 | 2 |
| Mali | 0 | 0 | 1 | 2 | 1 | 1 | 1 | 1 | 1 | 0 | 0 | 0 | 0 | 0 | 8 |
| Mauritania | 0 | 0 | 0 | 1 | 0 | 0 | 0 | 0 | 0 | 0 | 0 | 0 | 0 | 0 | 1 |
| Mozambique | 0 | 0 | 1 | 0 | 0 | 1 | 0 | 0 | 1 | 0 | 0 | 0 | 0 | 0 | 3 |
| Niger | 0 | 0 | 0 | 0 | 1 | 0 | 0 | 1 | 0 | 0 | 1 | 0 | 0 | 0 | 3 |
| Nigeria | 0 | 1 | 0 | 1 | 0 | 1 | 0 | 0 | 1 | 1 | 1 | 1 | 0 | 0 | 7 |
| Rwanda | 0 | 0 | 0 | 0 | 0 | 1 | 0 | 0 | 1 | 0 | 0 | 0 | 0 | 0 | 2 |
| Senegal | 1 | 1 | 2 | 1 | 1 | 0 | 1 | 1 | 0 | 0 | 0 | 0 | 0 | 0 | 8 |
| Sierra Leone | 0 | 0 | 0 | 0 | 0 | 0 | 1 | 0 | 0 | 0 | 0 | 0 | 0 | 0 | 1 |
| Somalia | 0 | 1 | 0 | 1 | 0 | 1 | 0 | 1 | 0 | 0 | 0 | 0 | 0 | 0 | 4 |
| Sudan | 1 | 1 | 1 | 1 | 1 | 1 | 2 | 1 | 1 | 0 | 1 | 0 | 0 | 0 | 11 |
| Togo | 0 | 0 | 0 | 1 | 0 | 0 | 0 | 1 | 0 | 0 | 0 | 1 | 0 | 0 | 3 |
| Uganda | 0 | 0 | 1 | 0 | 1 | 0 | 1 | 0 | 0 | 1 | 0 | 0 | 0 | 0 | 4 |
| United Republic of Tanzania | 0 | 3 | 1 | 2 | 2 | 2 | 1 | 1 | 1 | 2 | 1 | 0 | 2 | 0 | 18 |
| Zambia | 0 | 0 | 1 | 0 | 0 | 1 | 0 | 0 | 0 | 0 | 0 | 0 | 0 | 0 | 2 |

**Supplementary Figure 1: Mapping of ACT studies performed per country from 2010 to 2024 in sub-Saharan Africa**


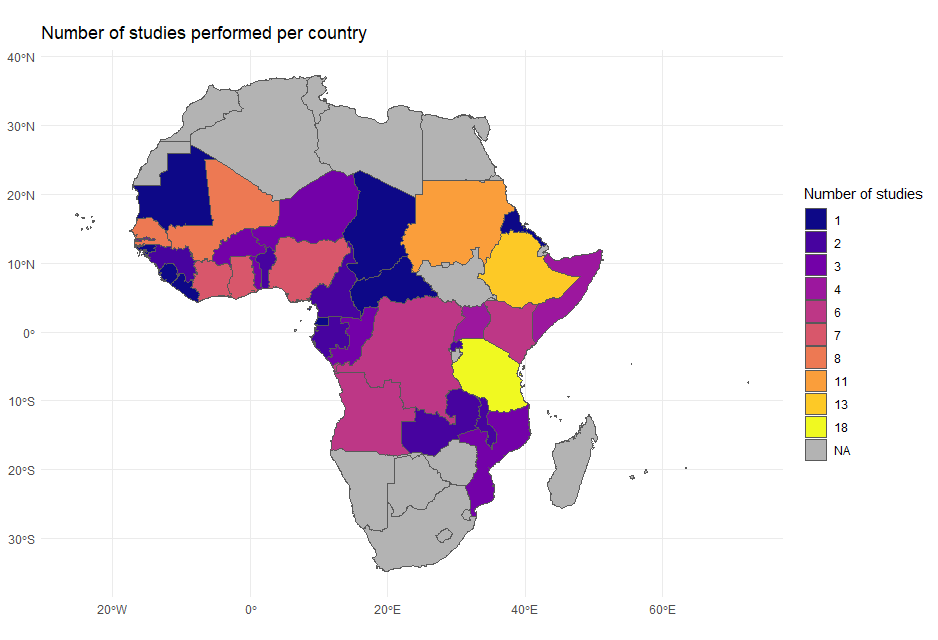


**Supplementary Figure 2: Evolution of ACPR corrected for ACT efficacy studies per country from 2010 to 2024 in North Africa**


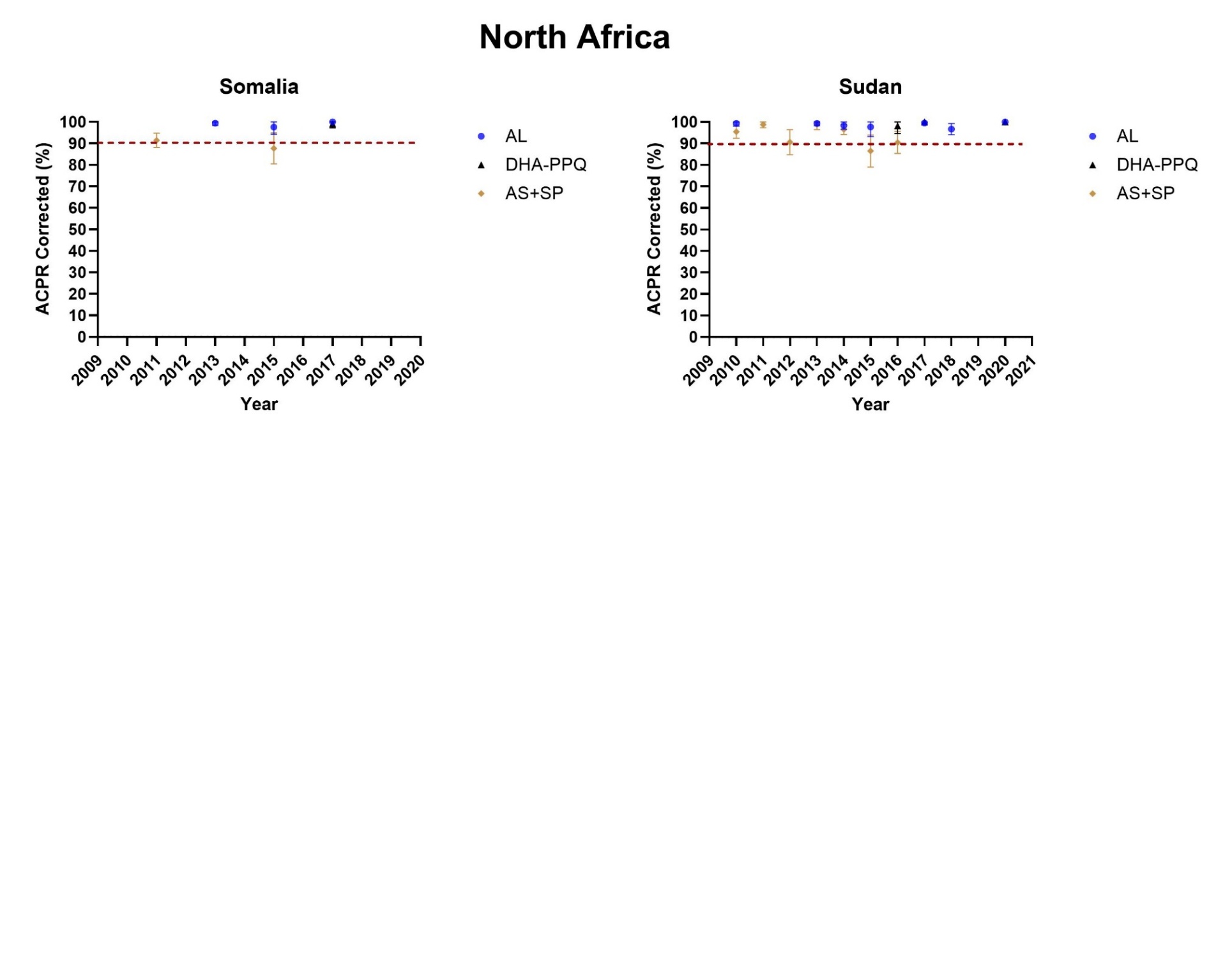


**Supplementary Figure 3: Evolution of ACPR corrected for ACT efficacy studies per country from 2010 to 2024 in West Africa**


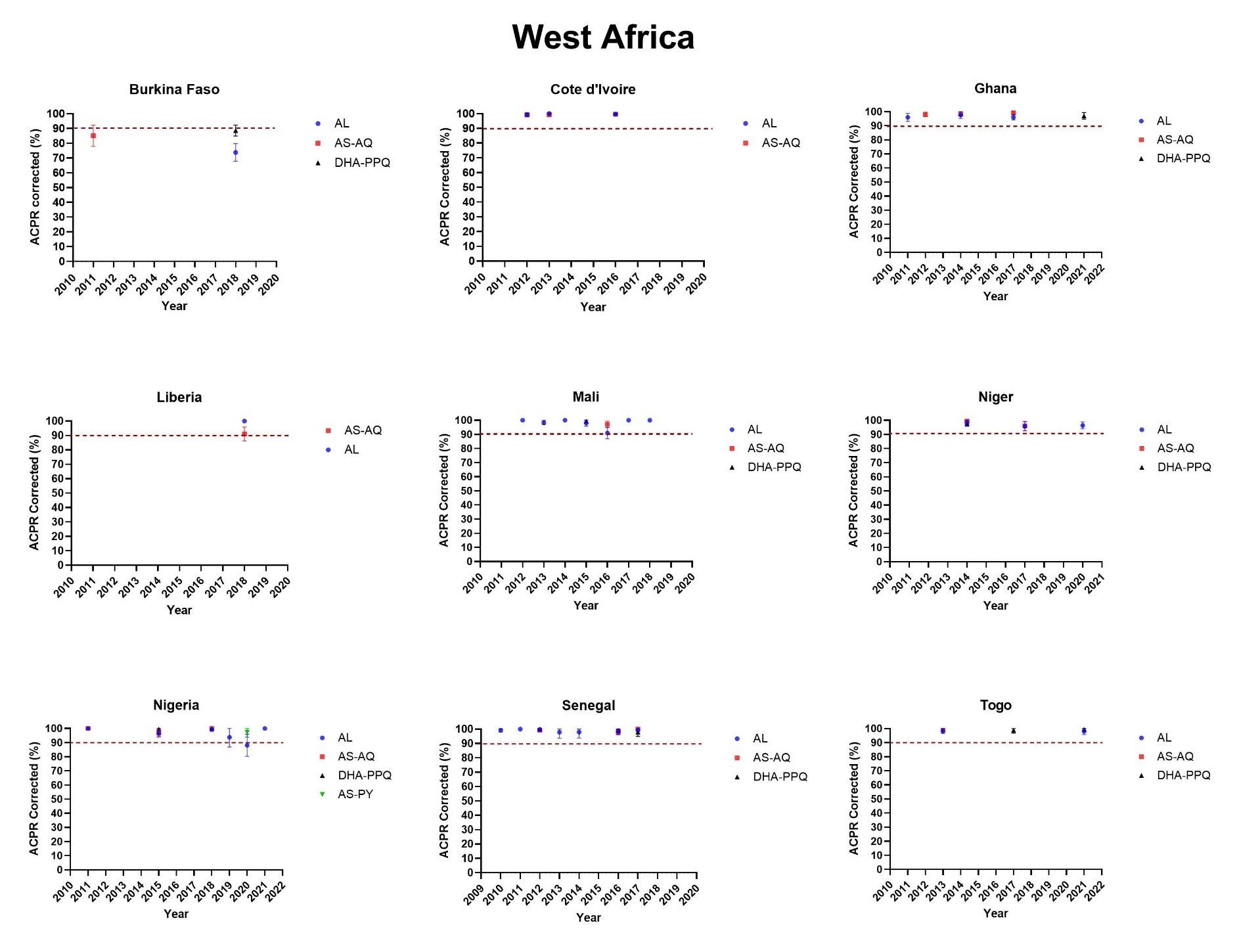


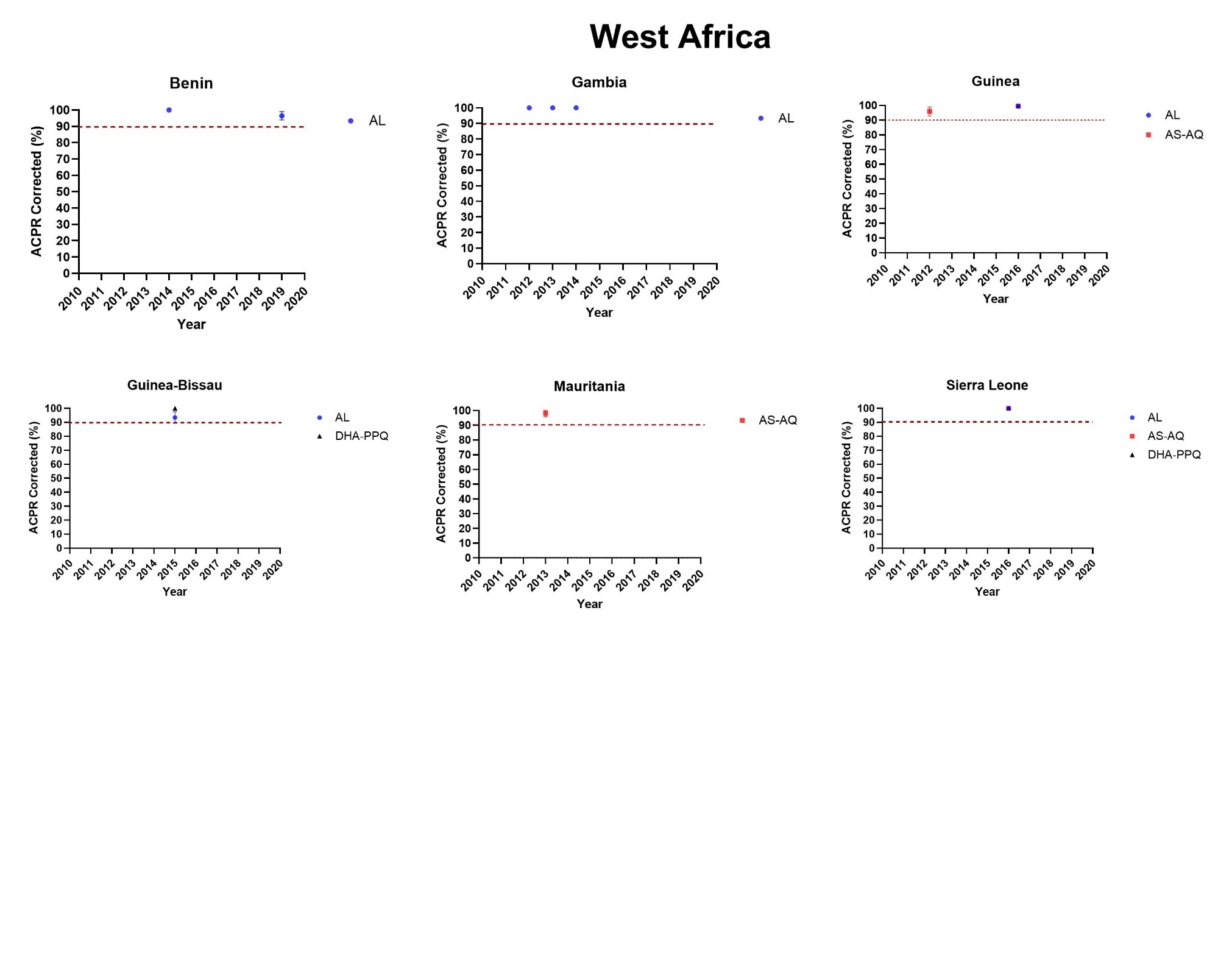


**Supplementary Figure 4: Evolution of ACPR corrected for ACT efficacy studies per country from 2010 to 2024 in Central Africa**


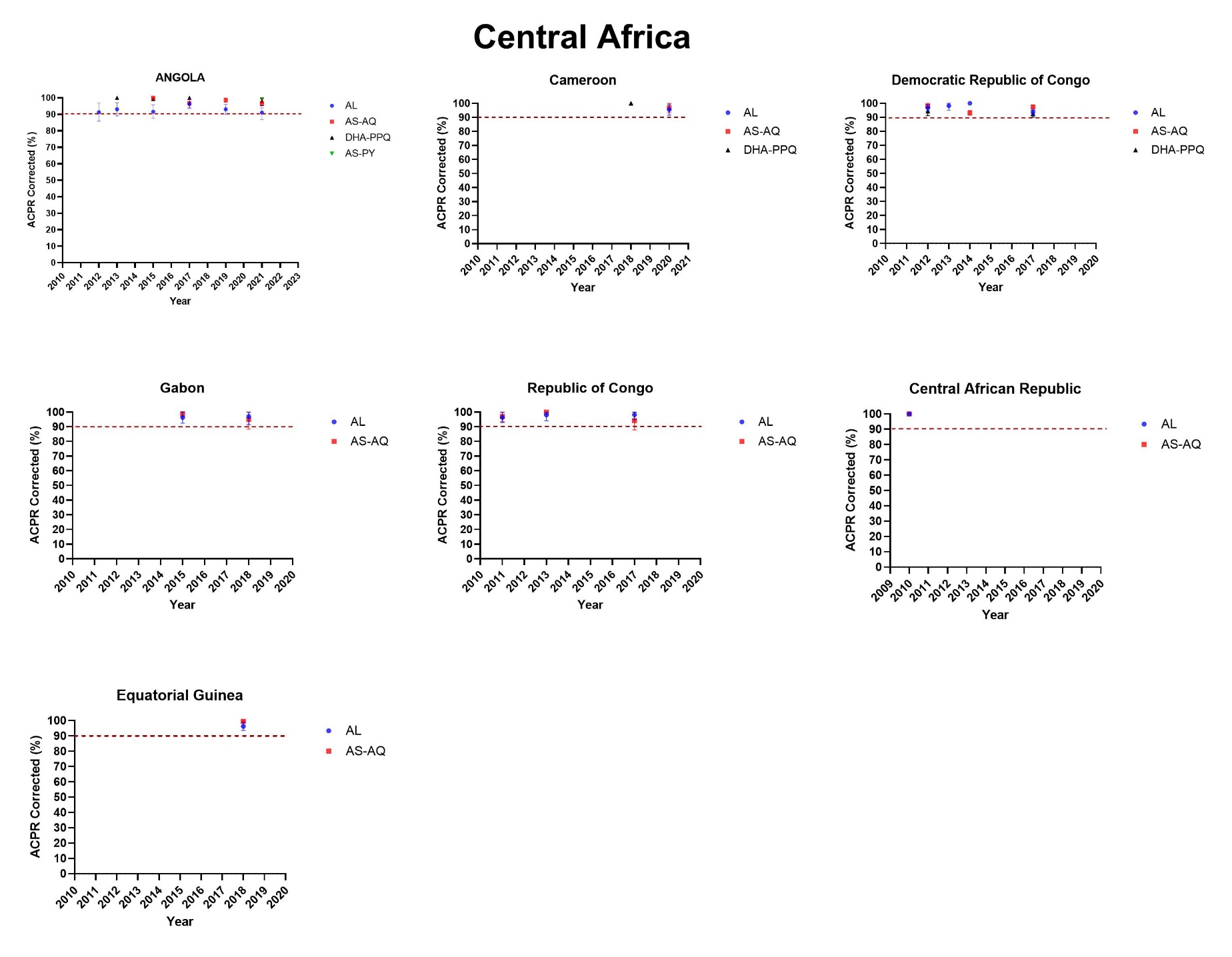


**Supplementary Figure 5: Evolution of ACPR corrected for ACT efficacy studies per country from 2010 to 2024 in East Africa**


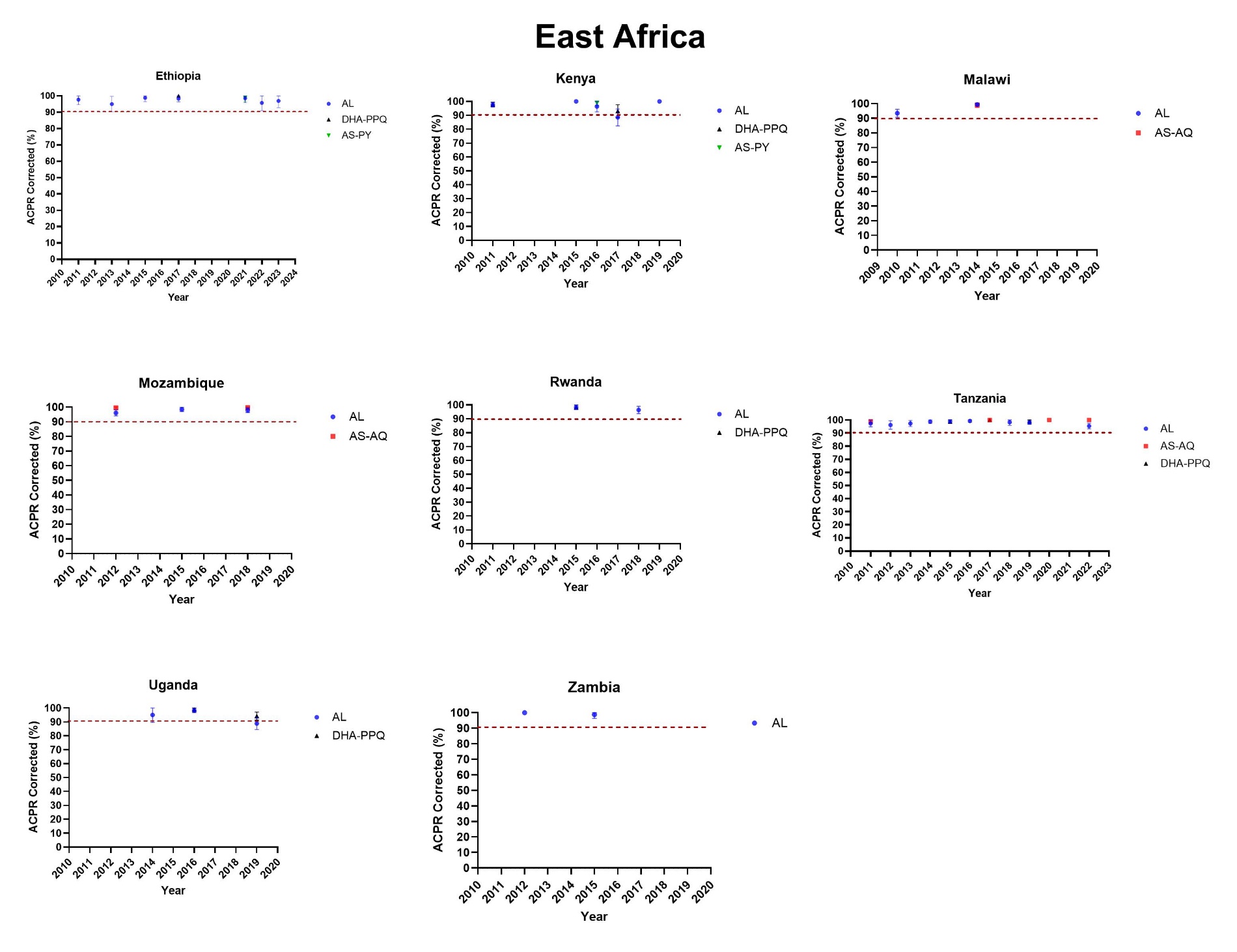

Supplement: Supplementary file 2 — Supplementary Material 2 [file 12879_2025_12130_MOESM2_ESM.docx]
